# Discrimination of *Panax ginseng* from counterfeits using single nucleotide polymorphism: A focused review

**DOI:** 10.3389/fpls.2022.903306

**Published:** 2022-07-28

**Authors:** Zheng Ying, Muhammad Awais, Reshmi Akter, Fengjiao Xu, Sul Baik, Daehyo Jung, Deok Chun Yang, Gi-Young Kwak, You Wenying

**Affiliations:** ^1^Weifang Engineering Vocational College, Qingzhou, China; ^2^Graduate School of Biotechnology, College of Life Sciences, Kyung Hee University, Yongin-si, South Korea

**Keywords:** single nucleotide polymorphism, saponins, landraces, cultivars, markers

## Abstract

Discrimination of plant species, cultivars, and landraces is challenging because plants have high phenotypic and genotypic resemblance. *Panax ginseng* is commonly referred to as Korean ginseng, which contains saponins with high efficacy on cells, and has been reported to be worth billions in agroeconomic value. Korean ginseng’s increasing global agroeconomic value includes additional species and cultivars that are not Korean ginseng but have physical characteristics close to it. This almost unidentifiable physical characteristic of Korean ginseng-like species is discriminated via molecular markers. Single nucleotide polymorphism (SNP), found across the plant species in abundance, is a valuable tool in the molecular mapping of genes and distinguishing a plant species from adulterants. Differentiating the composition of genes in species is quite evident, but the varieties and landraces have fewer differences in addition to single nucleotide mismatch. Especially in the exon region, there exist both favorable and adverse effects on species. With the aforementioned ideas in discriminating ginseng based on molecular markers, SNP has proven reliable and convenient, with advanced markers available. This article provides the simplest cost-effective guidelines for experiments in a traditional laboratory setting to get hands-on SNP marker analysis. Hence, the current review provides detailed up-to-date information about the discrimination of *Panax ginseng* exclusively based on SNP adding with a straightforward method explained which can be followed to perform the analysis.

## Introduction

**FIGURE 1:**
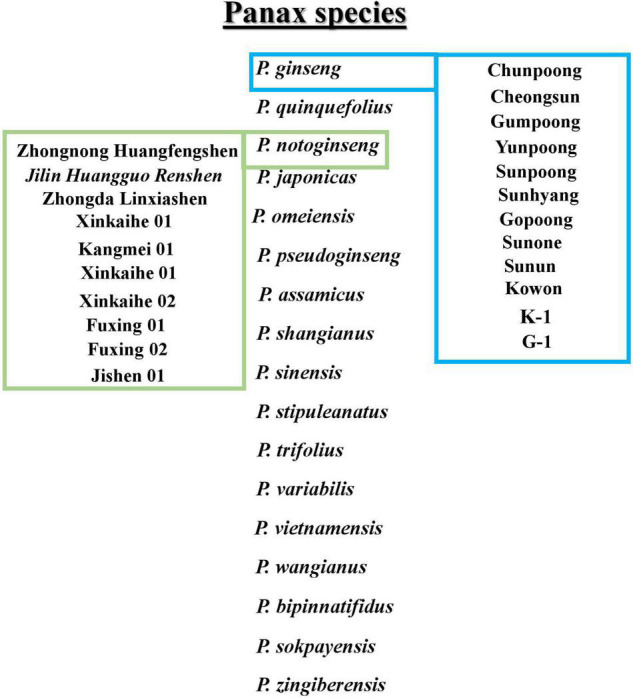
Description of *Panax* species. In boxes *Panax ginseng* and *Panax notoginseng* cultivars are mentioned.

Ginseng belongs to the genus *Panax* and the family Araliaceae, commonly known as Korean ginseng, and has excellent medicinal properties. It is widely used in East Asia as an herbal medicinal plant (Wang et al., 2019; Zhang et al., 2020). Local Korean and overseas consumers favor Korean ginseng (*Panax ginseng*). The favored Korean ginseng has a modern medical science verification of effectiveness in improving blood circulation and brain function (Kennedy and Scholey, 2003; Li et al., 2017), enhancing immune function (Rivera et al., 2005), preventing diabetes (Lai et al., 2006), and improving sexual performance (de Andrade et al., 2007), as well as having anticancer and anti-aging properties (Keum et al., 2000; Li et al., 2017). The other most used species of the genus *Panax* include *P. quinquefolius*, *P. notoginseng*, *P. japonicus*, *P. pseudoginseng*, *P. vietnamensis*, *P. omeiensis*, *P. assamicus*, *P. shangianus*, *P. sinensis*, *P. stipuleanatus*, *P. trifolius*, *P. variabilis*, *P. wangianus*, *P. bipinnatifidus*, *P. sokpayensis*, and *P. zingiberensis*. In addition, several cultivars are introduced mainly for enhancing saponins or protecting against root rot diseases in ginseng species. The Korean cultivars include Chunpoong, Yunpoong, Gopoong, Sunpoong, Gumpoong, Cheongsun, Sunhyang, Sunun, Sunone, K-1, G-1, and Kowon. Furthermore, Jilin Huangguo Renshen, Jishen, Fuxing 01, Fuxing 02, Kangmei 01, Xinkaihe 01, Xinkaihe 02, Zhongnong Huangfengshen, and Zhongda Linxiashen belongs to the Chinese cultivars (Zhang et al., 2020). The conclusive presentation of ginseng species and cultivars belonging to *Panax ginseng* and *P. notoginseng* is shown in Figure 1.

Korean ginseng has outstanding qualities compared to other species in the genus, primarily due to the higher content of ginsenosides. Korean ginseng is highly valued as it provides a basis for its higher price; however, such quality sometimes causes fraudulent labeling (Jung et al., 2014). Additionally, cultivars have been frequently mixed-cultivated on local farms alongside seeds of ginseng cultivars sold in the market by home seed producers. The complexities involved in Korean ginseng’s growth condition, such as the cultivation process, require a careful monitoring system in its cultivating condition, close to its natural habitat (Jo et al., 2017). If proper measures are not taken when growing the medicinal plants, it hinders the management of ginseng cultivation and affects the quality control of ginseng products (Wang et al., 2016a), safeguarding public health and consumers’ rights (Wang et al., 2011a). Though the medicinal values and economic benefits of different ginseng preparation processes are significantly different, product adulteration and substitution severely affect its quality and causes widespread problems in the ginseng market (Ichim and de Boer, 2020).

**FIGURE 2:**
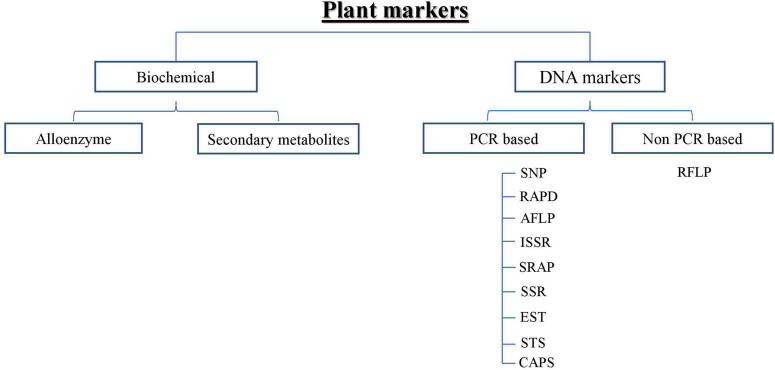
Schematic representation of Plant markers.

To overcome the problem of counterfeits, efficient discrimination methods for ginseng varieties/cultivars are necessary to combat adulterants. For the last few decades, traditional methods of discriminating ginseng cultivars have been based on phenotypic observations, but environmental and developmental factors often affect morphological characteristics. Different ginseng cultivars are highly similar in their morphology, especially in the early developmental stage of ginseng, dried ginseng, seeds, and seedlings. Hence, rendering differentiation is quite tricky and sometimes impossible. Besides, differentiating ginseng cultivars based on their morphological characteristics cannot be used to screen many ginseng samples. Therefore, a simple method of DNA analysis is straightforward and highly desirable (Wang et al., 2010a). Thus, techniques have been introduced in the form of allozymes (Koren et al., 2003), restriction fragment length polymorphism (RFLP) (Ngan et al., 1999), simple sequence repeats (SSR) (Bang et al., 2011), amplified fragment length polymorphism (AFLP) (Ha et al., 2002), random amplified polymorphic DNA (RAPD), polymerase chain reaction-restriction fragment length polymorphism (PCR-RFLP) (Um et al., 2001), and single nucleotide polymorphism (SNP) (Kim et al., 2016). A comprehensive description of biochemical and DNA markers is provided in Figure 2.

Though several review articles have been published on the discrimination of Ginseng (Jo et al., 2017; Goodwin and Proctor, 2019; Ichim and de Boer, 2020) that cover the overall genetic markers, a concise review article on SNP-based studies has not been published yet. Furthermore, regarding research articles, a high amount of data has been published on SNP in cpDNA, mtDNA, and nuclear DNA. Therefore, there is an immediate need for summarized literature to be published that wholly deals with the studies based on single nucleotide polymorphism (SNP). The current review article explains SNP-based discrimination of the ginseng species and cultivars step-by-step, i.e., starting from the chloroplast to mitochondria and then the nuclear genome. In addition, a simple method to perform the SNP analysis in traditional labs is provided.

## Single nucleotide polymorphism marker for the discrimination of *Panax ginseng*

**FIGURE 3:**
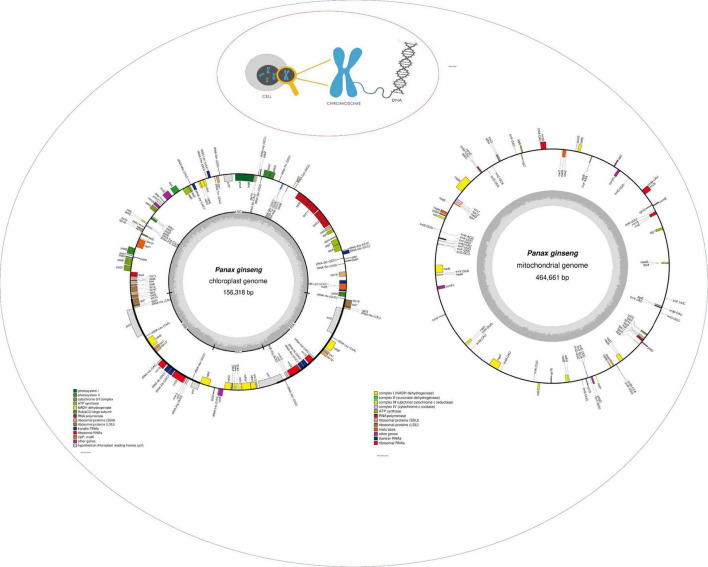
Representation of the typical genomes found in plant cell. The description of genes in cpDNA and mtDNA is highlighted in colors.

Figure 3 Illustration of the three genomes found in a plant cell. Chloroplast and mitochondrial genome are drawn with OGDRAW (Lohse et al., 2007).^1^

### Discrimination of *Panax* species based on Chloroplast DNA (cpDNA)

The chloroplast is photosynthetic organelles that provide sustenance to green plants. In angiosperms, most of the chloroplast genomes are circular, having duplex DNA, containing one large single-copy region (LSC), one small single-copy region (SSC), and a pair of inverted repeats (IRs). In chloroplast, gene content and order are highly conserved, but the genome size fluctuates from 120 to 160 kb in length. Most studies are concerned with plant species discrimination; evolutionary studies and phylogenetic relationships are based on cpDNA because of its haploid nature, maternal inheritance, highly conserved gene content, and genome structure (Song et al., 2017). Adding to it, cpDNA is sometimes highlighted as useful to identify species (Kim et al., 2017), though, in the case of *P. ginseng*, few studies have been performed. With cpDNA, the first-ever study to distinguish ginseng species, cultivars, and landraces with SNP and dCAPS markers (In dCAPS assay, a mismatch/or mismatches in PCR primer is used to create restriction endonuclease (RE)-sensitive polymorphism based on the target mutation) was conducted by Kim et al. (2015) using InDeL regions on tandem repeats. One of the InDeL at intergenic regions of *rps16-trnUUG* had two variants of tandem repeats, 13 and 33 bp, which identified one inbred Korean cultivar “Sunhyang” and *P. quinquefolius*, respectively, in comparison to other Korean ginseng cultivars. Another Tandem repeat with a size of 57 bp in the *ycf1* gene discriminated Chunpoong and Hwangsook from different *P. ginseng* cultivars and *P. quinquefolius*. In addition, the dCAPS method using restriction enzymes in the *rpoc2* gene was considered and, on the SNP site, with a cleavage, generated unique bands of Gumpoong and Cheongsun, among other cultivars; furthermore, *rpoc1* exon SNP was detectible by dCAPS marker and developed a unique amplicon for Chunpoong.

While continuing with the trend to distinguish ginseng species on the plastid genome, Nguyen et al. (2017) performed a comparative analysis of the five *Panax* species and developed gcpm1-14 markers that could discriminate the *Panax* species. Out of 14, 8 markers produced unique amplicon sizes and were specific to different *Panax* species. Among the 14 markers, 13 markers could not distinguish between the two cultivars of *P. ginseng*, i.e., Chunpoong and Yunpoong. Still, the gcpm12 discriminated between the two cultivars and produced an amplicon size of 316 and 373, respectively. Furthermore, gcpm3, gcpm8, and gcpm10 markers could distinguish *P. notoginseng* from the other *Panax* species, while the gcpm4 marker was specific to *P. quinquefolius*. The marker gcpm6 discriminated against *P. japonicus* while gcpm9 and gcpm14 discriminated against *P. vietnamensis* from the rest of the 4 *Panax* species. Among all the primer sets, gcpm12 was the most variable and produced amplicon sizes ranging from 202 (*P. vietnamensis*) to 316 (*P. ginseng*, Chunpoong).

**FIGURE 4:**
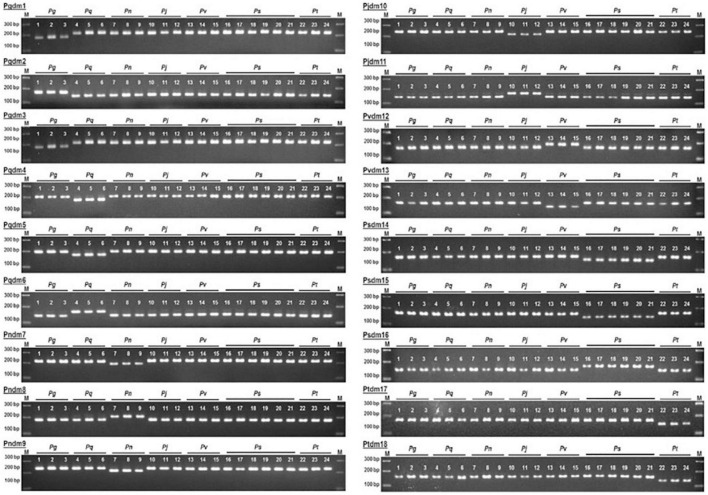
Representation of 18 SNP dCAPS markers for the discrimination of different *Panax* species. The image is obtained from Giang et al. (2020).

Kim et al. (2015) and Giang et al. (2020) used dCAPS method for differentiating ginseng. Basically, dCAPS is a modification of CAPS. It requires a restriction enzyme that cleaves the DNA at specific sites; research conducted by Giang et al. (2020), who did a comparative analysis of seven *Panax* species, showed the complete chloroplast genome was carried out and that 1,128 SNP in 71 coding gene sequences were identified, while the *psaJ*, *psbN*, *rpl23*, *psbF*, *psbL*, *rps18*, and *rps7* were identical among the seven species. Species delimitation was performed based on unique polymorphism found in the sequences found upon multiple alignments; 18 dCAPS primer pairs were designed. For the first two primer sets, Pgdm1-2 were designed on the genes *rpl20* and *ndhK* using the restriction enzyme Cla1. The 3rd primer pair was derived from *rps15* using the restriction enzyme Sma1 resulting in specificity for *P. ginseng* compared to other species. The markers Pqdm4-6 were specific to *P. quinquefolius* and were designed on the *rpoC1*, *ndhA*, and *ndhK* genes, digested by Sal1, Cha1, and Alu1, respectively. Pndm7-9 markers were obtained from the genes *rpoC1*, *rpoC2*, and *ndhK* digested with the help of Sal1 *Hin*dIII, and Cla1 made a unique pattern that was specific to *P. notoginseng*. For discriminating *P. japonicus*, markers Pjdm10-11 derived from *rpoC2* and *rpoB* sequences were cleaved with Sal1 and Rsa1, respectively. The markers Pvdm12 and 13, Psdm14-16, and Ptdm17 and 18 were able to discriminate the *P. vietnamensis*, *P. stipuleanatus*, and *P. trifolius* from the other *Panax* species successfully. Figure 4 describes the patterns of the bands via the gel-based electrophoresis. The genes discussed in the current article are summarized in Table 1.

**TABLE 1:** Summarized genes/regions used in the article.

Genome type	S. no	Gene name/Region	References
**Chloroplast genome**	1	rpl20, rps15, ndhK, rpoC2, ndhH, psbB, rpoB, ndhA, rpoC1	Giang et al., 2020
	2	rps16–trnQ-UUG, atpH–atpI, petA–psbJ, rpl14–rpl16, rps2–rpoC2, trnE–trnT, clpP–psbB	Nguyen et al., 2017
	3	*rps16-trnUUG, rpl32-trnUAG, ycf1, trnUUC-trnGGU, rpoc2, rpoc1*	Kim et al., 2015
	4	trnC-rps16, trnS-trnG, petB, trnE-trnM, psbM-trnD	Manzanilla et al., 2018
**Mitochondrial genome**	1	Mitochondrial NADH dehydrogenase subunit 7 (nad7) intron 3	
			Li et al., 2017
	2	Mitochondrial NADH dehydrogenase subunit 7 (nad7) intron 3	Wang et al., 2011b
	3	Mitochondrial nad7 intron 4	Wang et al., 2009
	4	Mitochondrial cytochrome oxidase subunit 2 (cox2) intron I and intron II	Wang et al., 2010a
	5	Mitochondrial cytochrome c oxidase subunit 2 (cox2)	Wang et al., 2016b
**Nuclear genome**	1	Pathogenesis-related protein 5 gene	Wang et al., 2019
	2	Auxin repressed protein gene	Kim et al., 2016
	3	Major latex-like protein (MLP-like) gene	Sun et al., 2010
	4	Dammarenediol synthase (DS)	Wen-Ru et al., 2020
	5	Dammarenediol synthase (DS)	Grazina et al., 2021
**Ribosomal DNA**	1	45S ribosomal DNA	Yang et al., 2017
	2	26S ribosomal DNA	Wang et al., 2010b
	3	ITS region, including 5.8S	Lee et al., 2010
	4	ITS region, including 5.8S	Kim et al., 2007
	5	External transcribed spacer (ETS)	Wang et al., 2011a
	6	ITS region 2	Osathanunkul and Madesis, 2019

For the specific species, the suitability of genetic markers is sometimes measured in interspecific distance to observe the number of variable sites or pairwise distance between sequences. Highlighted methods include the Refined Single Linkage (RESL) algorithm implemented in the Barcode of Life Database (BOLD) (Ratnasingham and Hebert, 2013) or OBITools (Boyer et al., 2016), etc., whereas a more nuanced technique, mPTP, is a tree-based method that enhances the quality of the selection process of the barcoding markers compared to traditional markers. Furthermore, the technique becomes more useful if some regions are carefully selected which highlight specific structural patterns that enable the discrimination of species, as in the case of ginseng species, i.e., *P. ginseng*, *P. quinquefolius*, *P. bipinnatifidus*, and *P. stipuleatus* whose identification is quite a complex process because of recently evolved plastid genome. Manzanilla et al. (2018) used the mPTP approach toward the determination of all the *Panax* species. The method includes the selection of suitable markers upon the extraction of SNP density over the whole plastid genomes and retrieved 2052 SNP. With further analysis, three regions were selected for barcoding analysis, each of the regions contain 83 SNP on average within the *Panax* species, and fifteen markers were designed. Out of 15 analyzed markers, only four (*trnC-rps16*, *trnS-trnG*, *petB*, and *trnE-trnM*) were able to discriminate most ginseng species. When used together, three markers (*trnC-rps16*, *trnE-trnM*, and *psbM-trnD*) confidently determine the most traded ginseng species, i.e., *P. ginseng*, *P. quinquefolius*, and *P. vietnamensis*. For further directions, confirming the result via gel-based method or real-time PCR, all the *Panax* samples with multiple accessions per taxon are to be made to observe variation in the selected markers.

### Discrimination of *Panax* species based on mitochondrial DNA (mtDNA)

In angiosperms, the mitogenome, compared to the chloroplast genome, was not used to analyze the phylogeny over several decades. In the recent past, researchers have been interested in the potential usefulness of mitochondrial data for phylogenetic data (Han et al., 2021). With regard to differentiating between species or cultivars, much work has been published on the mitogenome in ginseng. One of the genes present in Figure 3, mitochondrial NADH dehydrogenase subunit 7 (nad7) intron 3, was identified by Li et al. (2017) to discriminate between Russian wild ginseng, Chinese cultivated ginseng, and *P. ginseng* cultivars. With the help of a multi aligned sequence, it was identified that nad7 intron three domains have a homologous sequence between Russian wild ginseng, Korean cultivated ginseng, and Chinese cultivated ginseng, except that a single nucleotide polymorphism “G” was detected in Russian wild ginseng at 700 bp in comparison to Chinese cultivated ginseng “T” at the same position. Allele-specific PCR and real-time PCR were performed to obtain an optimized result for distinguishing the plant species. Furthermore, a research article by Wang et al. (2011b) used a multiplex amplification refractory mutation system (MARMS) to distinguish between “Gumpoong” and “Chungsun” from all of the other cultivars of *P. ginseng* using mitochondrial nicotinamide adenine dinucleotide (NADH) dehydrogenase subunit 7 (nad7) intron three region. Adding to it, (Wang et al., 2009) considered mitochondrial nad7 intron four region to discriminate Chunpoong cultivar by designing a modified allele-specific primer and using multiplex PCR.

Chunpoong has superior quality over other cultivars and gives a high yield and high resistance to ginseng rusty root rot disease. Wang et al. (2010a) discriminated Chunpoong using mitochondrial cox2 intron II region. A total of six primer pairs were used, from which two acted as universal to produce a 2,179-bp fragment and one acted as a control. In the case of Chunpoong, three primer sets, i.e., Forward, Reverse, and Specific, were designed and used to obtain a size of 1,037 bp. In addition to the other eight cultivars, a specific primer pair set was designed and gave an 1,183 bp band in size. To validate the results, a large number of ginseng samples were analyzed, and discrimination of the Chunpoong was obtained with accuracy.

The major ginseng cultivars cultivated in China, such as Damaya, Ermaya, Biaotiao, Changbo, and Huangguo, are mainly distinguished according to their phenotype. Damaya has higher medicinal and commercial value, often mixed with other cultivars for commercial purposes. To address the issue, Wang et al. (2016b) identified (cox2) as a marker to discriminate Damaya from other cultivars, Ermaya, Biantiao, Changbo, and Huangguo. Upon their sequencing alignments, the resulting sequences were utterly identical. The exception of SNIP (variation of single nucleotide at a specific position among the individuals) was found to be on the nucleotide position 386, replacing a nucleotide in cultivar Damaya A to C. Three primer pairs (forward, reverse, and specific) were designed. Allele-specific PCR was performed, which resulted in a 410 bp band specific to Damaya, while the other cultivars generated only a specific band of 771 bp.

As technology progresses, new techniques are being introduced into the scientific community. One such technique is Kompetitive Allele-Specific PCR (KASP), a newly developed method of single nucleotide polymorphism (SNP) genotyping, which requires only a few SNP markers to genotype various samples. The technique is set by the LGC Genomics Ltd., and is an efficient and low-cost genotyping method. The accuracy and convenience of the KASP assay have made it popular in the analysis of corn (*Zea mays*) and wheat (*Triticum aestivum*) (Ma et al., 2021). Furthermore, concerning the *P. ginseng* cultivar, Gumpoong assembly of the whole mitogenome was performed by Jang et al. (2020). In addition, using the technique, 10 SNP markers were designed to evaluate the diversity between 59 Korean ginseng accessions, including ten accessions of *P. quinquefolius* and *P. notoginseng*.

### Discrimination of *Panax* species based on nuclear DNA (nDNA)

#### Discrimination of *Panax ginseng* species/cultivars based on functional genes

The nuclear genome mainly contains functional genes for metabolic pathways, such as auxin repressed genes. These candidate genes can sometimes be used to identify the best cultivar. Accordingly, Wang et al. (2019) discriminated against the *P. ginseng* cultivar K-1, which is well known for its good root shape and thus suitable for producing red ginseng and productive lateral roots and effective disease resistance. Data mining of 5 pathogenesis-related (PR) proteins was performed; out of five, one gene PR5 (thaumatin-like protein) contained the SNP region at 289th position (A for G). Multiplex PCR and real-time PCR assays were applied to distinguish K-1 from the rest of the *P. ginseng* cultivars and 4 Chinese ginseng cultivars (Damaya, Ermaya, Biantiao, and Huangguo). With the help of a specific primer set, an exclusive 310 bp band was generated, which was unique to the K-1 cultivar, while a 577 bp common band was generated by using two universal primer pairs; it was used as a positive control to show that PCR conditions are well optimized. To determine whether the base substitution (A for G) in the K-1 cultivar is responsible for its disease resistance, bioinformatics tools were used, revealing that non-synonymous substitution leads to modifying amino acid residue from aspartic acid (D) to asparagine (N). Modification of the amino acid residue increased the instability index and isoelectric point of PR5 of K-1. The base substitution also changed the secondary structure, including the length of the coil and strand. Further, it changed the tertiary structure of the PR5 protein of K-1, which could be the reason for disease resistance in the K-1 cultivar.

One of the critical genes responsible for the growth and development of the plant is Auxin repressed genes (Park and Han, 2003). Kim et al. (2016) used Auxin repressed protein gene to distinguish the Chunpoong (*P. ginseng* cultivar) from *P. quinquefolius* and eight cultivars of Korean ginseng using modified allele-specific primer pair producing a specific 215 bp band. In contrast, on InDel region, a specific primer set for *P. quinquefolius* was designed, generating a specific band of 489 bp. The universal primer pair produced 609-bp amplicons that were common to all the ginseng cultivars and *P. quinquefolius*.

Sun et al. (2010) revived the work of Wang et al. (2009) as it was efficient on a limited range of cultivars. Sun et al. (2010) used the “major latex-like protein (MLP-like) gene” expressed highly in 4-year-old Chunpoong. An InDeL and SNP-based marker research was conducted. The InDel marker could identify hybrid F1, which is cross-cultivated between *P. ginseng* and *P. quinquefolius.* The SNP-based marker helped in analyzing the Chunpoong population or plant breeding program based on real-time PCR. The genes discussed in the current article are summarized in Table 1.

The worth of *P. ginseng* is primarily due to ginsenosides, which are saponins. Some researchers like Wen-Ru et al. (2020) considered both the metabolomics and molecular techniques to distinguish the ginseng species and cultivars. In the case of molecular discrimination, functional genes like Dammarenediol synthase (DS), which is the critical functional enzyme in the cyclization of 2, 3-oxidosqualene to dammarenediol II, are the first step of skeleton formation for the most bioactive ginsenoside. Upon the multi align, a sequence of *P. ginseng* and *P. quinquefolius* was found, two SNIPS in the intron region, and designed two specific primer sets to discriminate between *P. ginseng* and *P. quinquefolius* and gave 571 and 341 bp bands, respectively. Furthermore, the method is reliable and sensitive as it can generate results only with 0.1 ng of DNA. Dammarenediol synthase (DS) enzyme is involved in the biosynthetic pathway of ginsenosides. In addition, ginsenoside’s profile varies within the species and genus with temperature, region, and age. Taking this into consideration, Grazina et al. (2021) hypothesized DS gene as a potential marker and discriminated five major ginseng species, namely *P. ginseng*, *P. quinquefolius*, *P. notoginseng*, *P. japonicus*, and *P. trifolius*, using high resolution melting (HRM) analysis. In addition, the applicability of the method developed was effective for commercial ginseng products that include herbal infusions, dried roots, and plant food supplements.

### Discrimination of *P. ginseng* species and cultivars in nrDNA, ITS, and 5.8S region

According to the China plant BOL group, the internal transcribed spacer ITS region has higher discriminatory power than the plastid genome (Li et al., 2015). Furthermore, Yang et al. (2012) confirmed that the ITS region provides geographic information about the plant species as a molecular marker. In addition, the ITS region is a good selection for a marker to determine phylogeny at the interspecific and intergeneric levels among flowering plants and other eukaryotes. In contrast, a specific primer set designed in the divergent ITS regions can be used to authenticate the original plants from counterfeits (Feng et al., 2010). Continuing with the trend to identify species based on ITS region, (Osathanunkul and Madesis, 2019) used the combination of a DNA barcode with HRM to discriminate *P. ginseng* from *P. notoginseng*. Additionally, Thai ginseng (*Talinum paniculatum*) and *Phytolacca americana* were differentiated from Korean *ginseng* due to their strong resemblance in root shape. Initially, three primer sets were selected, ITS2, *rbcL*, and *trnL*, to simultaneously identify the best among them for discrimination of all selected species. *rbcL* primer pair generated a unique melting curve for *Talinum* and *Panax* species, while no distant curves were obtained for *Phytolacca* species. While in the case of *trnl* melting curves for *P. americana*, *P. japonica*, *P. ginseng*, and *P. notoginseng* were nearly identical. ITS2 region, on the other hand, had distant melting curves for all the species and determined all the species successfully. The reference diagram is provided in Supplementary Figure.

Yang et al. (2017) analyzed the 45S region of Korean ginseng cultivar and *P. quinquefolius*, a few snips were exclusive to G-1 and *P. quinquefolius.* The Exclusive primer set for G-1 and *P. quinquefolius* yielded a band in size of 449 bp. In contrast, the specific primer pairs for *P. quinquefolius* produced a band size of 255 bp. In contrast, the positive control band size for all the ginseng cultivars was 562. The experiments were performed ten times to validate the results. Wang et al. (2010b) adopted the Multiplex PCR method to discriminate the Gumpoong (*P. ginseng* cultivar) from other cultivars. Also, *P. notoginseng*, *P. quinquefolius*, and a few Chinese cultivars such as Biantiao Damaya and Ermaya use the 26S rDNA region.

**FIGURE 5:**
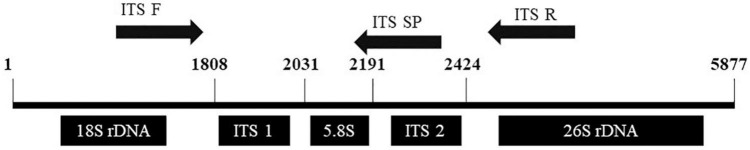
Representation of 18S, ITS 1, 5.8S ITS2, and 26S in rDNA of *Panax ginseng*.

Over the last 10 years, nucleic acids have been widely altered by substituting the phosphodiester linker or the sugar-phosphodiester backbone with various neutral or charged structures. More than a few of these modified oligonucleotides have developed properties in terms of affinity and binding to DNA and RNA. One of the significant representatives of this new type is peptide nucleic acids (PNAs) (Pellestor and Paulasova, 2004). Lee et al. (2010) used the technology of PNAs to identify between *P. ginseng*, *P. quinquefolius*, and *P. japonicus* using 5 PNA probes designed on 3 SNP in the 5.8S ITS region. Furthermore, a signal intensity comparison between PNA and DNA microarrays was performed to show PNA microarray provides a significantly more stable and specific fluorescent signal intensity than the DNA microarray. With the trend in the identification of *P. ginseng* based on the ITS region, Kim et al. (2007) identified several SNP in ITS and the 5.8S region of rDNA in Korean ginseng cultivars, and two of the ginseng species, *P. quinquefolius*, and *P. japonicas*, that were cleaved by Taq 1 polymerase at specific sites for discrimination of the species and cultivars. This discrimination method provides the breeders with basic information on target-specific cultivars. Figure 5 covers the region from 18S to 26S rDNA. The location of a specific primer pair on the ITS1 region is visualized since specific primer set position could differ among the desirability and regions of SNIP.

The external transcribed spacer (ETS) lies in the intergenic spacer region, which separates the repetitive 18S–5.8S–26S ribosomal gene blocks from each other (Poczai and Hyvönen, 2010). Wang et al. (2011a) upon multi alignment sequence of *P. ginseng* and *P. quinquefolius* exploited the two SNP in the ETS region. For both the species, specific primer sets were designed to have an intentional mismatch for more specificity. The remaining two forward and reverse primers were used as universal. A multiplex PCR analysis revealed *P. ginseng* with 388 bp and *P. quinquefolius* with 201 bp band sizes. Furthermore, mixed samples of both species and capsules, tea, and processed ginseng were also tested, which were identified efficiently, proving the method was reliable. The Supplementary File contains the Universal primer set position and specific primers pairs position for all the genes discussed in the current article.

## Working with single nucleotide polymorphism analysis

Here, we provide the simplest and most reliable method for working with the single nucleotide polymorphism (SNP). SNP are found in plant species across three genomes: the nuclear genome, mitochondrial genome (mtDNA), or chloroplast genome (cpDNA) (Li et al., 2015) explained and discussed the barcodes in genomes for the discrimination of plant species from a basic to newly developed system. But, most researchers themselves decide which regions they want to target and why they choose the specific region, as in the case of Wang et al. (2019) who used targeted functional genes “pathogenesis-related protein 5 gene” or Sun et al. (2010) who used “Major Latex-Like Protein Gene.” Likewise some researchers prefer to use mitochondrial genome or otherwise Chloroplast genome is preferred to discriminate the plant species, cultivars, and varieties.

**FIGURE 6:**
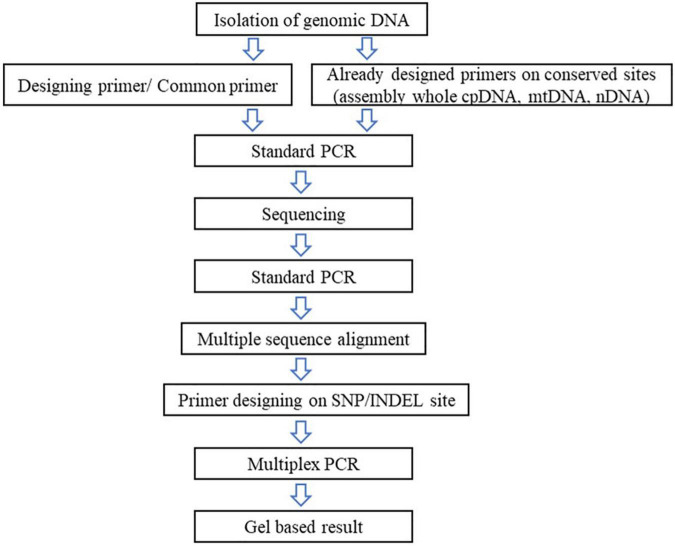
Schematic presentation to work with single nucleotide polymorphism.

Starting with genomic DNA Isolation is mainly done using CTAB (Doyle and Doyle, 1987), modified CTAB (Allen et al., 2006), or the DNA isolation kit method. The quantity and quality had to be ensured as it affects PCR reaction. Most researchers use 5 to 50 ng of DNA to perform PCR analysis. The next step to follow is the PCR with the universal primer set; if the plant cpDNA/mtDNA/nDNA is already available at NCBI^2^ or other available sites^3^ the target gene can be taken into the consideration and designing of the universal primer pair is brought about.^4^ The sequence data can be multi aligned with the help of online software.^5^ Multiple sequence alignment provides a better view to observe the SNIP or InDeL region between two or more species. A specific primer set design is brought about on the SNP site or InDeL region. Optimizing the Multiplex PCR is tedious as researchers work with 3–6 primer pairs in one reaction to optimize them with a single reaction condition. The schematic representation to work with analysis is shown in Figure 6.

This would be different, however, if a plant is chosen whose online data regarding genomes are not available, as in the case of *Terminalia ferdinandiana*, an Australian native plant commonly known as Kakadu plum plant found in Australia (Mohanty and Cock, 2012). In such cases, some researchers have already designed universal primer pairs on conserved regions, as Dong et al. (2013) who designed 138 sets of primers on the conserved regions on the plastid genome. Such primer pairs can be used to start identifying the genes along with the heterologous sites between related species. Table 2 provides only the primer sets with an amplification rate of 100%. According to Li et al. (2015), on the one hand, the whole-plastid-barcodes have great success in species distinguishing the closely related taxa and are cost-efficient, while standard technology is not available to most laboratories. However, researchers may have to choose between cost efficiency or particularity. In traditional labs, working with a single gene would be cost-effective and less time-consuming.

**TABLE 2:** Set of universal primer pairs according to Dong et al. (2013).

S. no	Sequence 5′ to 3′ (Forward, Reverse)	Locus	Type	Product size	Amplification success rate
1	GCGCATGGTGGATTCACAAATC	trnH	IGS	929	100
	TGCATGGTTCCTTGGTAACTTC	psbA			
2	TTACGTTCATGCATAACTTCCATACC	psbA	Exon	989	100
	AGAGAGACGCGAAAGCGAAAGCC	psbA			
3	GAAACAGGTTCACGAATACCAT	psbA	IGS	954	100
	CTTTCTTGTGCTAGAACTTTGGC	matK			
4	TCTAGCACACGAAAGTCGAAGT	matK	Exon	935	100
	CGATCTATTCATTCAATATTTC	matK			
5	AGTACTCTACCGTTGAGTTAGCAAC	trnK	IGS	918	100
	AATCGTTGCAATTGATGTTCGATCCC	rps16			
6	CTGGGACGGAAGGATTCGA	trnQ	IGS	1,272	100
	ATTGCTAATCCGTTGTACGAGTTA	trnS			
7	AAACTCTTCGTTTACACAGTAGTGA	psbI	IGS	1,112	100
	CTTTTACCACTAAACTATACCCGC	trnG			
8	GCGGGTATAGTTTAGTGGTAAAAG	trnG	Intron	1,306	100
	TTAGAAATTGGACAGGTAAAGAA	atpA			
9	GATTCCAAATTCAGAGCAATGCCTA	atpA	Exon	668	100
	AAGTGATTTATTAGATAATCGAAAAC	atpF			
10	ACACCAAGCACTACACTTAGAT	atpF	IGS	765	100
	ATGAATCCACTGATTTCTGCTGC	atpH			
11	AACAAAAGGATTCGCAAATAAAAG	atpH	IGS	1,544	100
	AGTTGTTGTTCTTGTTTCTTTAGT	atpI			
12	ATGATGGCCCTCCATGGATTCGCC	atpI	Exon	740	100
	ATGAATGTTCTATCATGTTCCATC	atpI			
13	GAACCTAATAAGATAGCAATTAC	atpI	IGS	980	100
	CGTAAAGGTATTCATATTAC	rps2			
14	AGATCCGGGTCACAATTTGTATC	rps2	Exon	1,785	100
	CTTGGGCAGTTTATTTGTGAAAATG	rpoC2			
15	GTCTTGGTCCCAATTCAAAA	rpoC2	Exon	830	100
	TTATTAGCAAAGAGGCGAAGAAA	rpoC2			
16	AAGCAAGAATACTATTTCTACG	rpoC2	Exon	950	100
	GGATCTGTCAATTATGTTATGG	rpoC2			
17	GTATGAAAAGTTCTTAATGTTA	rpoC2	Exon	1,003	100
	AATCTGGTCTTTCACAATAAAGTGATAGAT	rpoC2			
18	CCTAATGAAATAGATGTAGCAGTGGC	rpoC2	Exon	525	100
	GAAGCTCCTATCGAAGTTCATTATG	rpoC1			
19	TGTAGGGCTTCTTCGATTTCTCG	rpoC1	Exon	589	100
	GCTATTTGTTTACATCCATTAGTTT	rpoC1			
20	TACATAGAGTCCAATAAGCATATC	rpoC1	Exon	1,160	100
	CCCACTTTCTTACGATTACGAGGT	rpoC1			
21	GATCGGCTAATTGTTCTCGGATAG	rpoC1	Intron	1,346	100
	GATCGGTATAAACATCAAC	rpoC1			
22	TCGATAATTTCCACAAGCACAAAT	rpoC1	Exon	1,213	100
	TCGTATGCCCCGGAAGATAGATTA	rpoB			
23	ACTTTGATTTCACGTTTCTG	rpoB	Exon	955	100
	CCAGCTCGATACCGTCAAGAATT	rpoB			
24	GAACTCATTAAAGCCCGATT	rpoB	Exon	862	100
	GGAAGGATTGGTCGACAAAATATG	rpoB			
25	ATCCGCTACAGAACGAATACGTT	rpoB	Exon	925	100
	CAATACCTGGATTTAATCAGATAC	rpoB			
26	GGCGGCATGGCCGAGTGGTAAGGC	trnC	IGS	825	100
	TCCACTTCTTCCCCATACTACGA	petN			
27	ATGGATATAGTAAGTCTCGCTTGG	petN	IGS	1,326	100
	ATGGAAGTAAATATTCTTGCAT	psbM			
28	TTTGACTGACTGTTTTTACGTA	psbM	IGS	1,369	100
	GTTCAAATCCAGCTCGGCCCA	trnY			
29	CTGACCAATTGAACTACAATCCC	trnD	IGS	1,266	100
	AGCCCCTTATCGGATTTGAA	trnT			
30	GCCCTTTTAACTCAGTGGTAGAG	trnT	IGS	1,496	100
	CCAAATAGGAACTGGCCAATC	psbD			
31	ATGACTATAGCCCTTGGTAAATTTACC	psbD	Exon	1,058	100
	AAGAGCGTTTCCACGGGGTAGAACC	psbD			
32	CTCTTCTTCCAAGGGTTTCATAAT	psbD	Exon	1,158	100
	TATATCTTCCAAATCGTCCACACT	psbC			
33	ATGAAAACCTTATATTCCCTGAGGAGG	psbC	Exon	1,422	100
	GTTAAGAGGGGTCATGGAAAGAACAGG	psbC			
34	GAAGCAATCAAGAAAGCCGCATA	psaB	Exon	1,194	100
	TTCATTTTCAATTAGGCCTTGCT	psaB			
35	TGAATTACTATTCTCATTAA	psaB	Exon	1,520	100
	ATGGCATTAAGATTTCCAAGGTTTAG	psaB			
36	CATAAAGATTCCACTGACCTGTAA	psaB	Exon	904	100
	TGGCAAGAACTTATTGAATCCATCG	psaA			
37	TCCTACTGCAATAATTCTTGCTAAG	psaA	Exon	1,075	100
	TGTGGATTGGTGGATTTCTCATAG	psaA			
38	CATATCTTGAGGACGCCCTAA	psaA	Exon	1,403	100
	ATGATTATTCGTTCGCCGGAACCAG	psaA			
39	GTATGGCTATCGAAATCGTGAGCA	psaA	IGS	1,085	100
	ACCGGGGAGAACAGGCCATTCA	ycf3			
40	CGCCTCGTGATCTTCAACCAATT	ycf3	Intron	1,153	100
	ATGTCGGCTCAATCCGAAGGAAATT	ycf3			
41	AATGACAGATCACGGCCATATTATT	ycf3	Intron	1,086	100
	CCTAGATCGCGGATAAATGGAAA	ycf3			
42	TCAAATCGCACCATCTCTATAATAGGT	ycf3	IGS	1,531	100
	CCAAAACATTTGACTCTTCAC	rps4			
43	AGTCTGACGGGAATAATATTCTACGAC	rps4	Exon	602	100
	ATGTCGCGTTACCGAGGGCCTCGTTTC	rps4			
44	GTTCAAGTCCCTCTATCCCCA	trnL	IGS	1,281	100
	GATTTTCAAGAACGGGAATCTTA	ndhJ			
45	ATGAGCATCTTGAATTTCATAAAAA	ndhJ	Exon	1,675	100
	ATGTTTCTGCTTTATGAATATGATA	ndhC			
46	AGACCATTCCAATGCCCCCTTTCGCC	ndhC	IGS	1,466	100
	GTTCGAGTCCGTATAGCCCTA	trnV			
47	TAGGGCTATACGGACTCGAAC	trnV	Intron	876	100
	CCGCCGTATGAAAGCAATACTCTAA	trnM			
48	ACGAGTTGCTCTACCAACTGAGCTA	trnV	IGS	917	100
	ATGACCTTAAATCTTTGTGTACTGA	atpE			
49	ATAGCCTCAACTCGTGTCCTAGCTCG	atpE	Exon	689	100
	GTTACAAAGAACTTCAGGACAT	atpB			
50	ATGAGTTGGCGATCAGAACATATATGG	ycf4	Exon	545	100
	TTCAATTGGTACACGCAAGAAATAGG	ycf4			
51	GTTTCCACTTTTCCAGTCATT	cemA	IGS	488	100
	GCTTCTCGTGGATTTTCATA	petA			
52	AACAGATTACTCGATCCATTTCC	petA	Exon	924	100
	AAATTCATTTCGGATAATTGAACC	petA			
53	GGATTTGGTCAGGGAGATGC	petA	IGS	1,343	100
	ATGGCCGATACTACTGGAAGG	psbJ			
54	AGGGATGAACCCAATCCGGA	psbJ	Exon	762	100
	ATGTCTGGAAGCACAGGAGAACG	psbE			
55	ATCTACTAAATTCATCGAGTTGTTCC	psbE	IGS	1,490	100
	TATCTTGCTCAGACCAATAAATAGA	petL			
56	ACTAGTTATTTCGGTTTTCTA	petL	IGS	821	100
	AGGGATGTAGCGCAGCTTGGT	trnP			
58	GCAGGTCTATTGATAGAGATTAATCG	psaJ	IGS	1,170	100
	CCAGCAGTTCTAGTGGTCGACTCGGTT	rps18			
59	GCCAATCGGGGGATCGAATTGATTATAG	rps18	Exon	820	100
	GAATTAAACGAGGATATATAGCTCGG	rpl20			
60	GCTTGGGCTTCTCTTGCTGACAT	clpP	Intron	1,129	100
	TCCTAATCAACCGACTTTATCGAG	clpP			
61	GGGAATGCTAGACGTTTGGTAATTTC	clpP	Intron	1,829	100
	TGAATTGGTTATTCCTAAACGAGT	psbB			
62	ATGGGTTTGCCTTGGTATCGTGTTC	psbB	Exon	1,517	100
	TTGTCTTCTTGTAGTTGGATCTCC	psbB			
63	GAATTAGATCGTGCTACTTTGAA	psbB	Exon	998	100
	CATTGCGACACCCATCAAAGGA	psbH			
64	ATTAATACAATAGGATTTATGGTTAC	psbH	Intron	1,297	100
	CGATAGTAAAAAGTCATAGCAAA	petB			
65	AAAGTCTATGATTGGTTCGAAGAACG	petB	Exon	637	100
	AAAGGACCAGAAATACCTTGCTTACG	petB			
66	CAATCCACTTTGACTCGTTTT	petB	Intron	1,245	100
	GGTTCACCAATCATTGATGGTTC	petD			
67	ACCTGACTTGAATGATCCTGTATTAAG	petD	Exon	457	100
	GACCTAAAGTTAGGGATTTATCAATA	petD			
68	GAGAATGTTAATAAATTCCAAAA	petD	IGS	529	100
	TATTTTTATTGACCAATCAGA	rpoA			
69	TGTTTTCTCACGTTTTCGAT	rps8	Exon	1,208	100
	CCCAAAAGAACCAGATTCCGTAAA	rpl16			
70	AAATATCCAAATTTTGATGCC	rps3	Exon	641	100
	ATGGGACAAAAAATAAATCC	rps3			
71	TTCTACTTCTTCTTCCAAGTGCAGG	rpl2	Intron	1,440	100
	ATGGCGATACATTTATACAAAACTTC	rpl2			
72	TGGTTACGATTCTACCATATA	rpl2	Exon	1,069	100
	GTAGGATACTCCAAATTCGGG	ycf2			
73	TTCACTCTATCAATAACCGAGCCGG	ycf2	Exon	1,158	100
	GAAATGGTTTCACGGGATTCGGCCAA	ycf2			
74	TTCAACGAGATAGTGCTTTTTCAA	ycf2	Exon	971	100
	CGCTATGAGTTAGACTCAATAGAA	ycf2			
75	TATTCTTGTTATTGCTTCGACTC	ycf2	Exon	1,011	100
	GATCCGCTTGCCCCGAAATGACC	ycf2			
76	GGGTATCCTGAGCAATTGCAATAATC	ndhB	Exon	1,452	100
	AATGGACTCCTGACGTATA	ndhB			
77	GCAACGACTGGAGTGGGAGA	ndhB	Exon	889	100
	ATGATCTGGCATGTACAGAATGAAAAC	ndhB			
78	GATCATCAGAAGAAGAATTAGGCC	ndhB	Intron	1,475	100
	TGTCACGTCGAGGTACTGCAGAAG	rps7			
79	ACGAAAATGTGCAAAAGCTCTATTTG	rps7	Intron	1,171	100
	AAGGGTTAAGGATTTACCCGGTGTGA	rps12			
80	TTACTCCGACAGCATCTAGGGTTCC	rps12	IGS	1,884	100
	GGGATAATCAGGCTCGAACTG	trnV			
81	GAGTGTCACCTTGACGTGG	trnV	IGS	453	100
	CTCCTCAGCCTACGGGGTA	rrn16R			
82	TCTCATGGAGAGTTCGATCCTGGC	rrn16R	Exon	1,490	100
	AAAGGAGGTGATCCAGCCGCACC	rrn16R			
83	ATTCGTTCCCGGGCCTTGTAC	rrn16R	Intron	1,334	100
	CTCTACCAACTGAGCTATATCCCC	trnA			
84	CTGGTTCAAGTCCAGGATGGCCCA	trnI	Intron	1,138	100
	TCGGGAATCTCCGGATCTACGC	rrn23			
85	GGGTGATCTATCCATGACCAGGAT	rrn23	Exon	721	100
	CACCTGTTGTCCATCGACTAC	rrn23			
86	AAAACCTAAGGGTTCCTCCGC	rrn23	Exon	769	100
	AAGCTTCATAGGGTCTTTCTGTCC	rrn23			
87	CACAGGTCTCCGCAAAGTCGTAAG	rrn23	Exon	990	100
	TGGGCTTACTACTTAGATGCTTTC	rrn23			
88	CGGTGTGGGCGTTATAGCATTGA	rrn23	Exon	1,059	100
	ACCGTGGTTCGTAGCCACGTGCT	trnR			
89	TATTCTGGTGTCCTAGGCGTAGAGG	rrn5	IGS	1,104	100
	TCCTCAGTAGCTCAGTGGTAGAG	trnN			
90	CTCCCCAAGTAGGATTCGAAC	trnN	IGS	1,086	100
	GGTAATAAATAAGAGAATACTAAAGA	ycf1			
91	CGCGATTTATTTACATACTCGAAC	ccsA	IGS	592	100
	ACGTCAGATGTTCTATGGATACAA	ndhD			
92	ATATTCTTCAGTCATTGATAAACAA	ndhI	Exon	785	100
	TTTTATGTTGCTTCCTATCTAAAT	ndhA			

## Conclusion and perspectives

To detect the polymorphism within the plant species, cultivars, and varieties, a plethora of markers are readily available and used for the discrimination. A low mutation rate can be detected when it comes to SNP analysis. Furthermore, the outcome of the results is much swifter. As technology has been on the rise in the last few decades, the SNP analysis in discrimination has become recognizably important in herbal medicine. In particular, when it comes to *P. ginseng*, the most valuable medicinal herb, the estimated market worth is $2,084 million (Baeg and So, 2013). As the current article discusses distinguishing between the ginseng species, cultivars, and varieties, few reports discuss functional genes and how one cultivar acts better than the other. Some researchers have outlined a phenomenon known as horizontal plastid genome transfer into the mitochondrial and nuclear genomes (MTPTs) or nuclear genome sequences of plastid origin (NUPTs). These complexities in the genome can cause confusion, false results, and misidentification (Park et al., 2020). Though the work concerning the problem has already started, much more has to be done in the field. Fortunately, there are articles available online for *P. ginseng* cpDNA, mtDNA, and nDNA (see text footnote 2). So, dealing with MTPTs or NUPTs becomes easier as the researcher does not have to begin from scratch.

As the data above proves, most researchers have focused on nDNA or mtDNA regarding discrimination in ginseng plants. The mtDNA, along with cpDNA, is maternally inherited, and the evolution rate compared with the nuclear genome is much slower. It is well-established that the Mito genome is much larger. In addition, the protein-coding genes are fewer, as mentioned by Cui et al. (2021). In comparison ginseng plastid genome is much smaller which is easy to assemble furthermore, functional or protein-coding genes are higher in number. The above article discusses discrimination of ginseng on the functional genes in mtDNA and nDNA but in case cpDNA the gap is to be filled.

This review article summarizes the work on the discrimination of *P. ginseng* with the typical three genomes found in plants. It provides a concise work chart on working with SNP as a reliable method. The discrimination of *P. ginseng* via reliable SNP analysis is suitable for all plant species with convenience and cost-effectiveness that gives a chance to traditional labs to work with confidence in distinguishing plant species.

## Author contributions

MA and ZY wrote, drew figures and tables, and collected data of the manuscript. RA, FX, SB, and DJ contributed to collect the data. G-YK performed editing and helped with tables and figures. DY and YW conceptualized the manuscript. All authors contributed to the article and approved the submitted version.

## Footnotes

1
http://ogdraw.mpimp-golm.mpg.de/

2
https://www.ncbi.nlm.nih.gov/

3
https://www.arabidopsis.org/

4
https://primer3.ut.ee/

5
http://multalin.toulouse.inra.fr/multalin/
