# Supplementary material for: Discrimination of Panax ginseng from counterfeits using single nucleotide polymorphism: A focused review
Source: Front Plant Sci. 2022 Jul 28;13:903306. doi: 10.3389/fpls.2022.903306 (PMC9366256; doi:10.3389/fpls.2022.903306)
Supplement: Supplementary file 1 [file Presentation_1.pptx]

## Slide 1
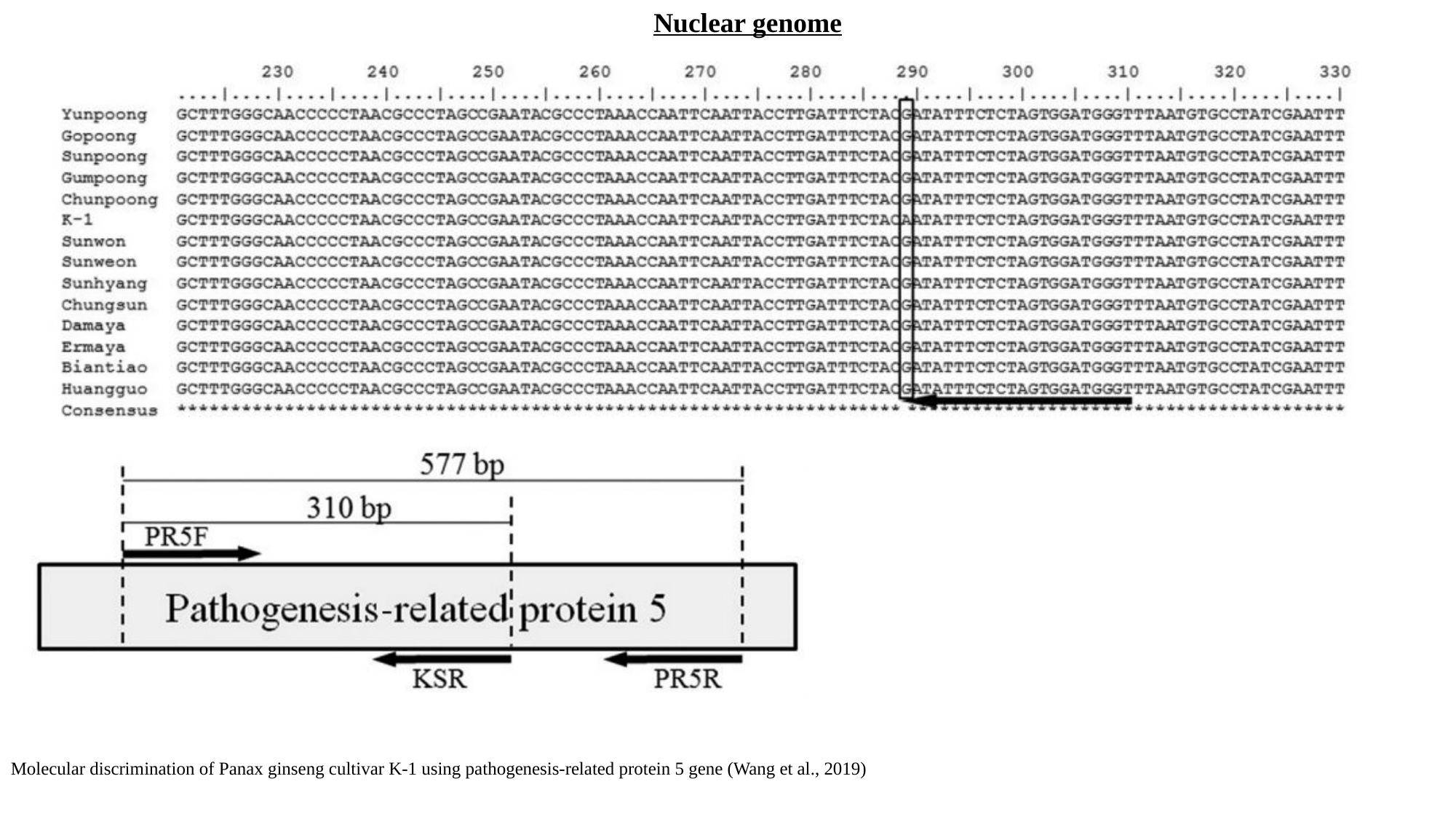

Nuclear genome
Molecular discrimination of Panax ginseng cultivar K-1 using pathogenesis-related protein 5 gene (Wang et al., 2019)

## Slide 2
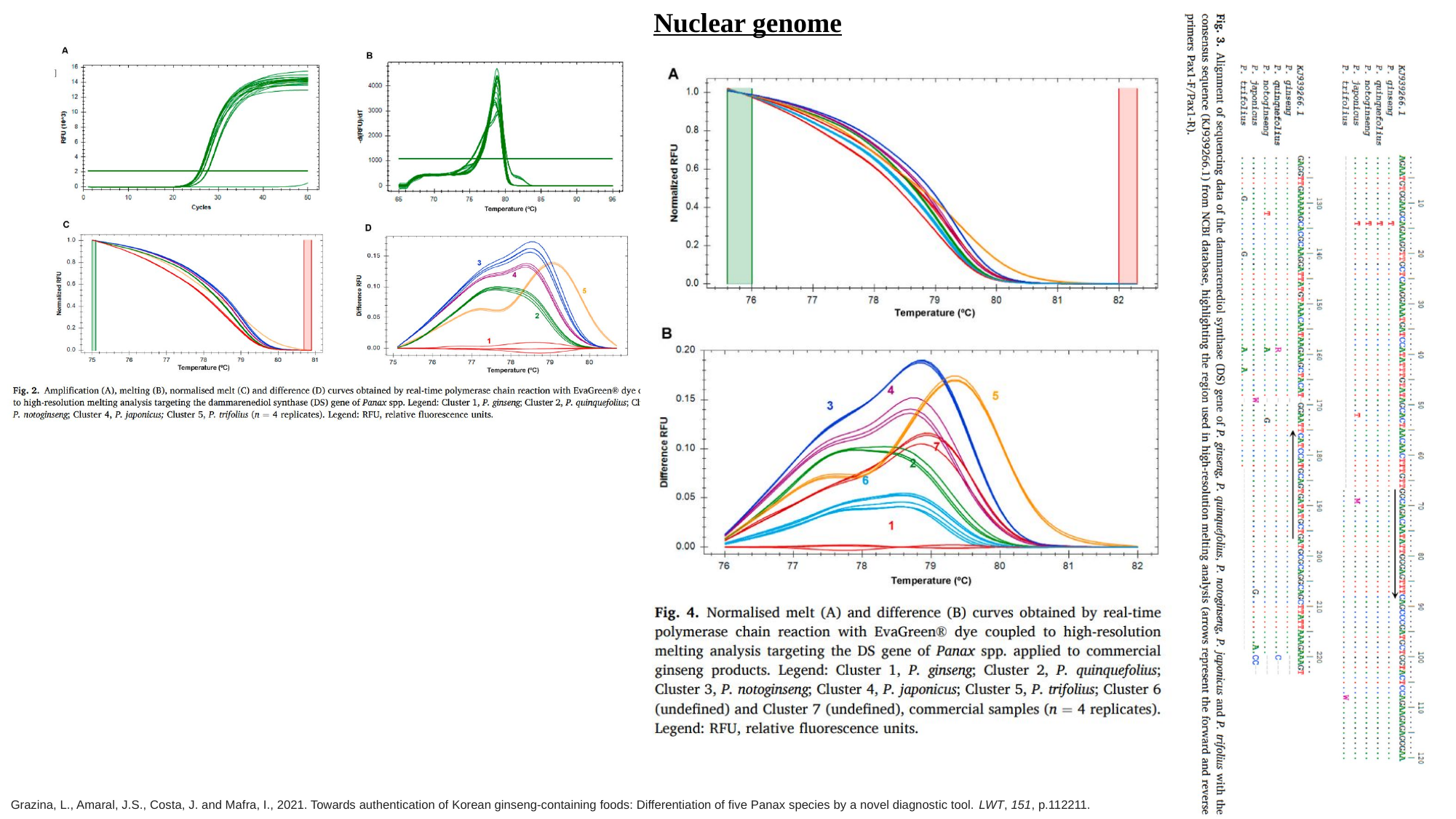

Nuclear genome
Grazina, L., Amaral, J.S., Costa, J. and Mafra, I., 2021. Towards authentication of Korean ginseng-containing foods: Differentiation of five Panax species by a novel diagnostic tool. LWT, 151, p.112211.

## Slide 3
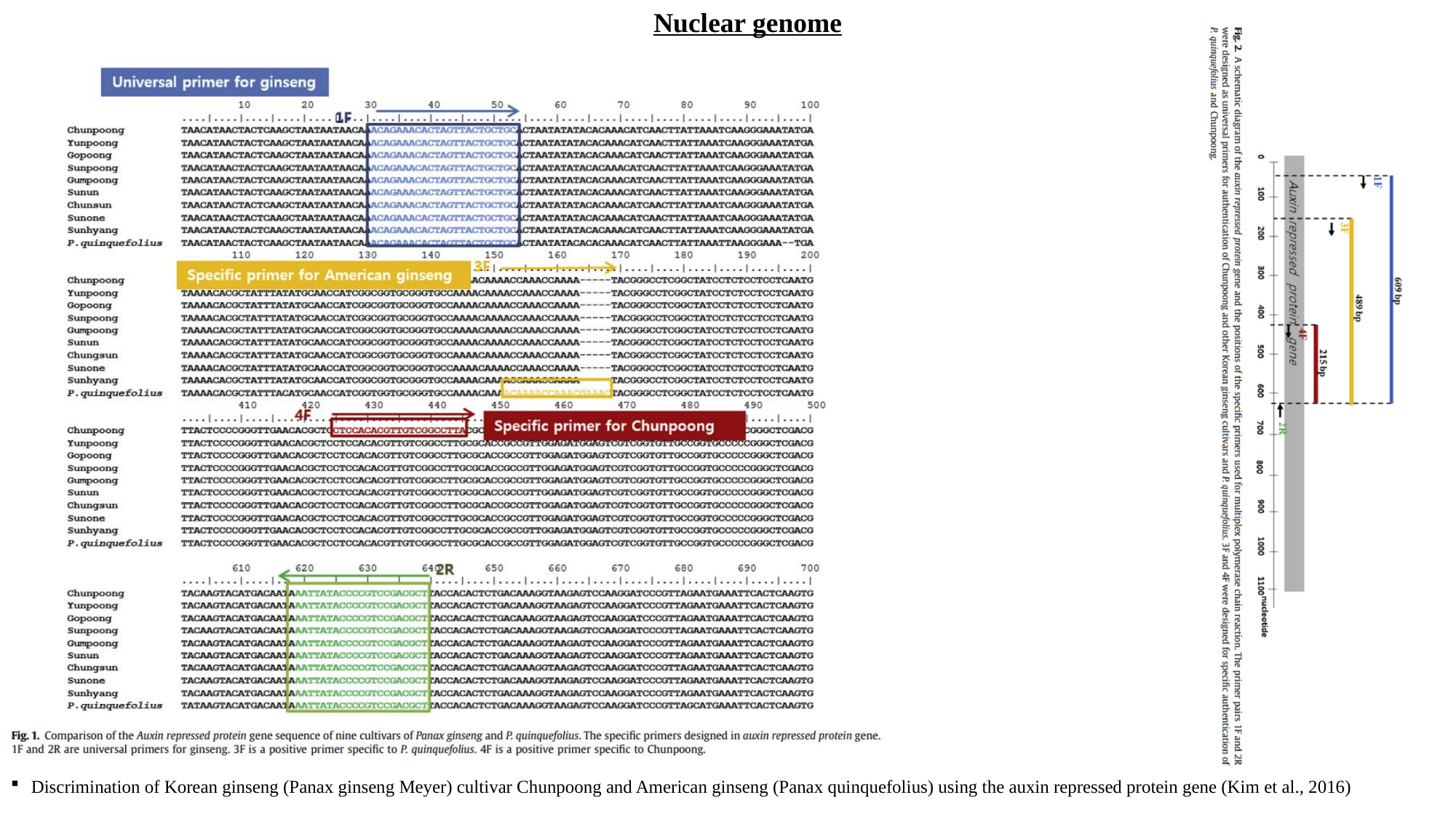

Nuclear genome
Discrimination of Korean ginseng (Panax ginseng Meyer) cultivar Chunpoong and American ginseng (Panax quinquefolius) using the auxin repressed protein gene (Kim et al., 2016)

## Slide 4
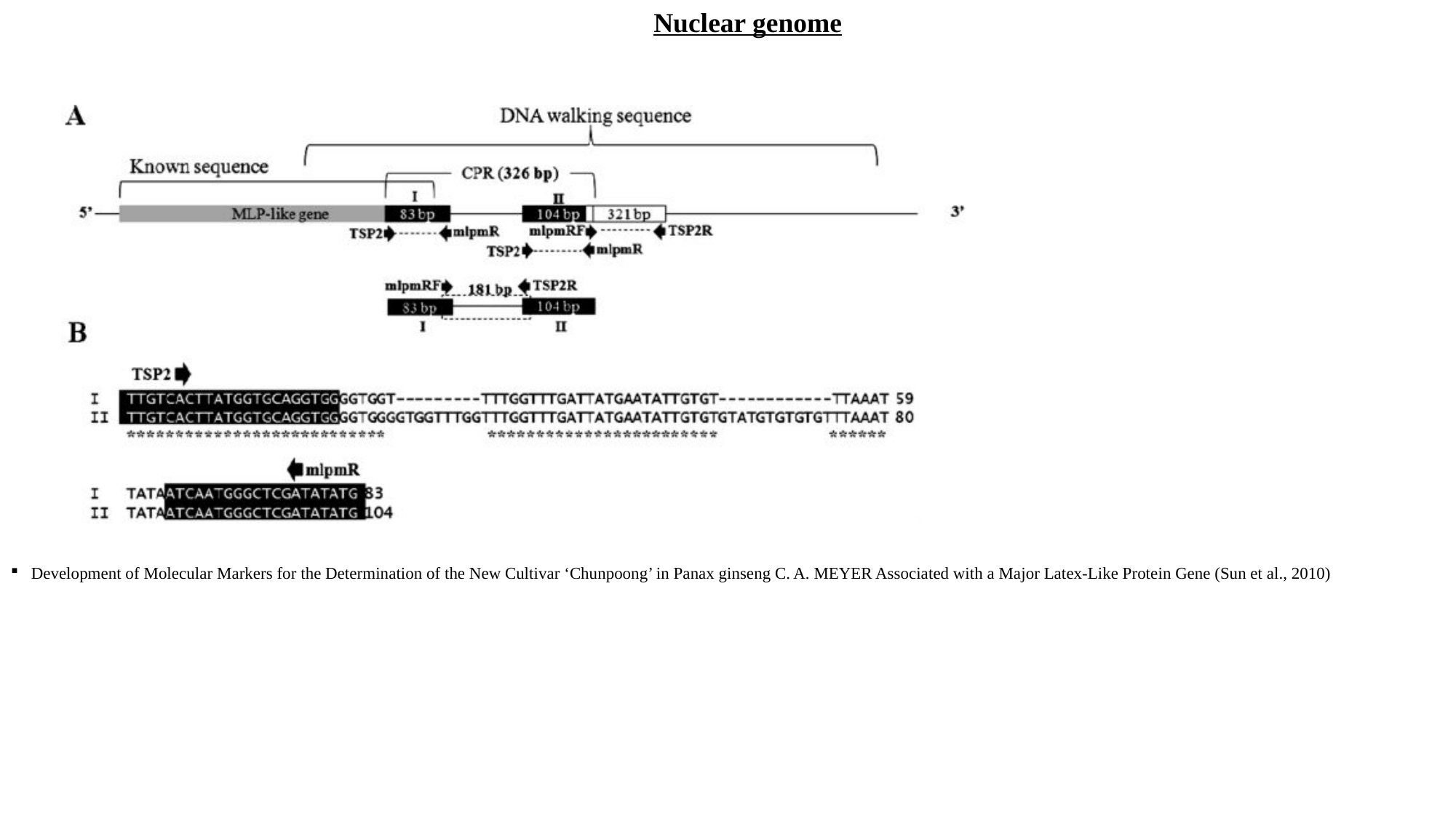

Nuclear genome
Development of Molecular Markers for the Determination of the New Cultivar ‘Chunpoong’ in Panax ginseng C. A. MEYER Associated with a Major Latex-Like Protein Gene (Sun et al., 2010)

## Slide 5
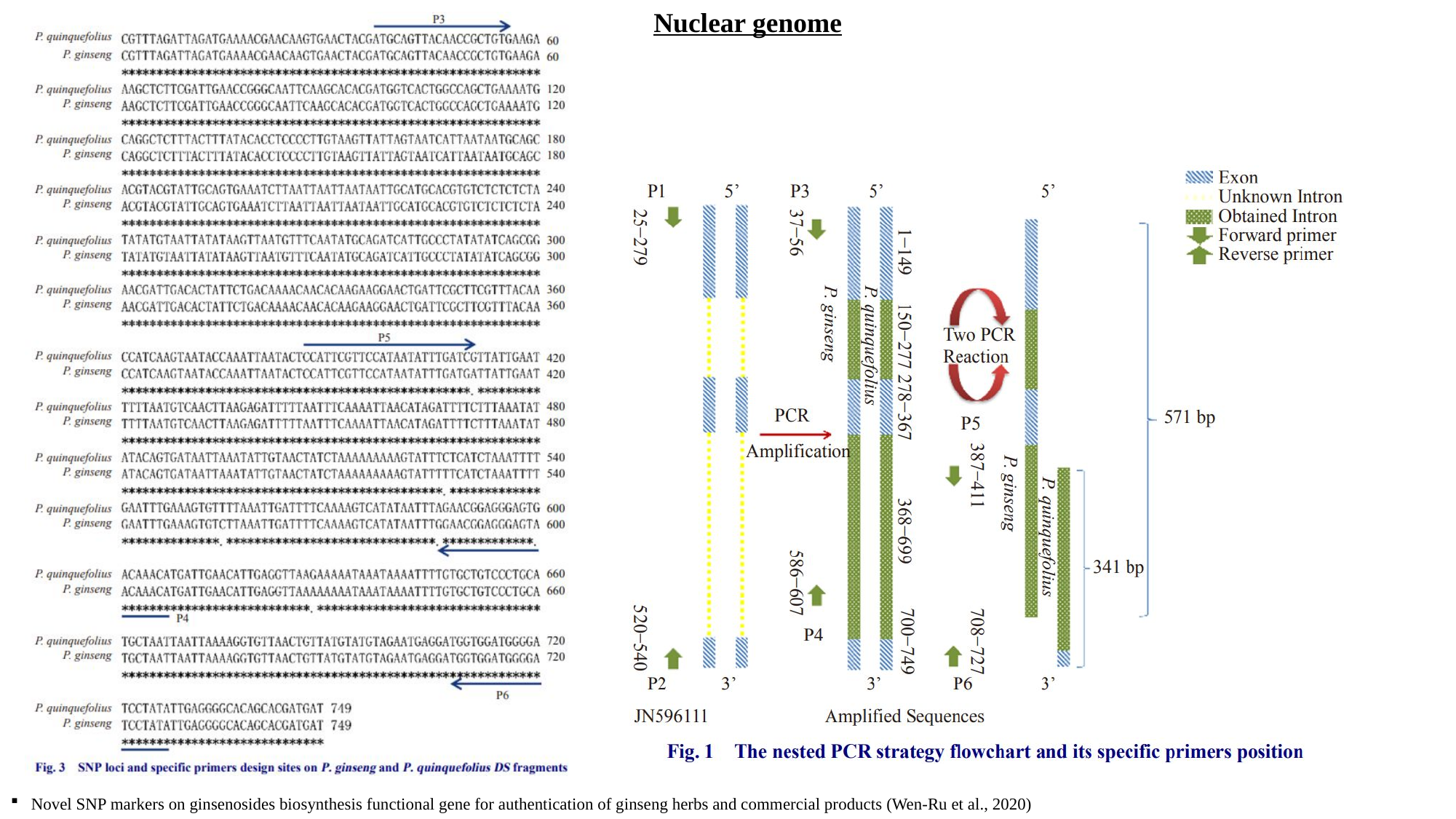

Nuclear genome
Novel SNP markers on ginsenosides biosynthesis functional gene for authentication of ginseng herbs and commercial products (Wen-Ru et al., 2020)

## Slide 6
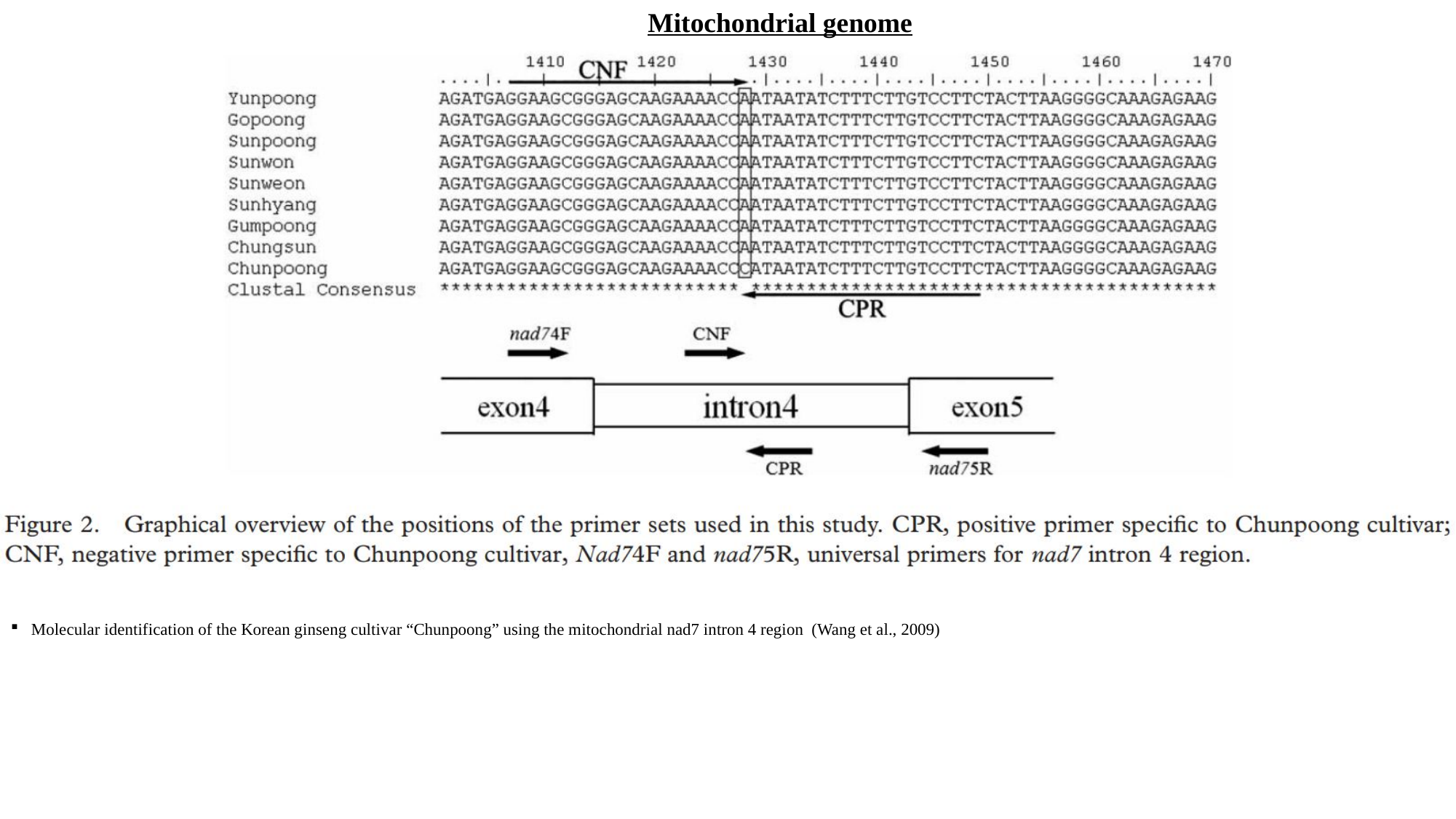

Mitochondrial genome
Molecular identification of the Korean ginseng cultivar “Chunpoong” using the mitochondrial nad7 intron 4 region (Wang et al., 2009)

## Slide 7
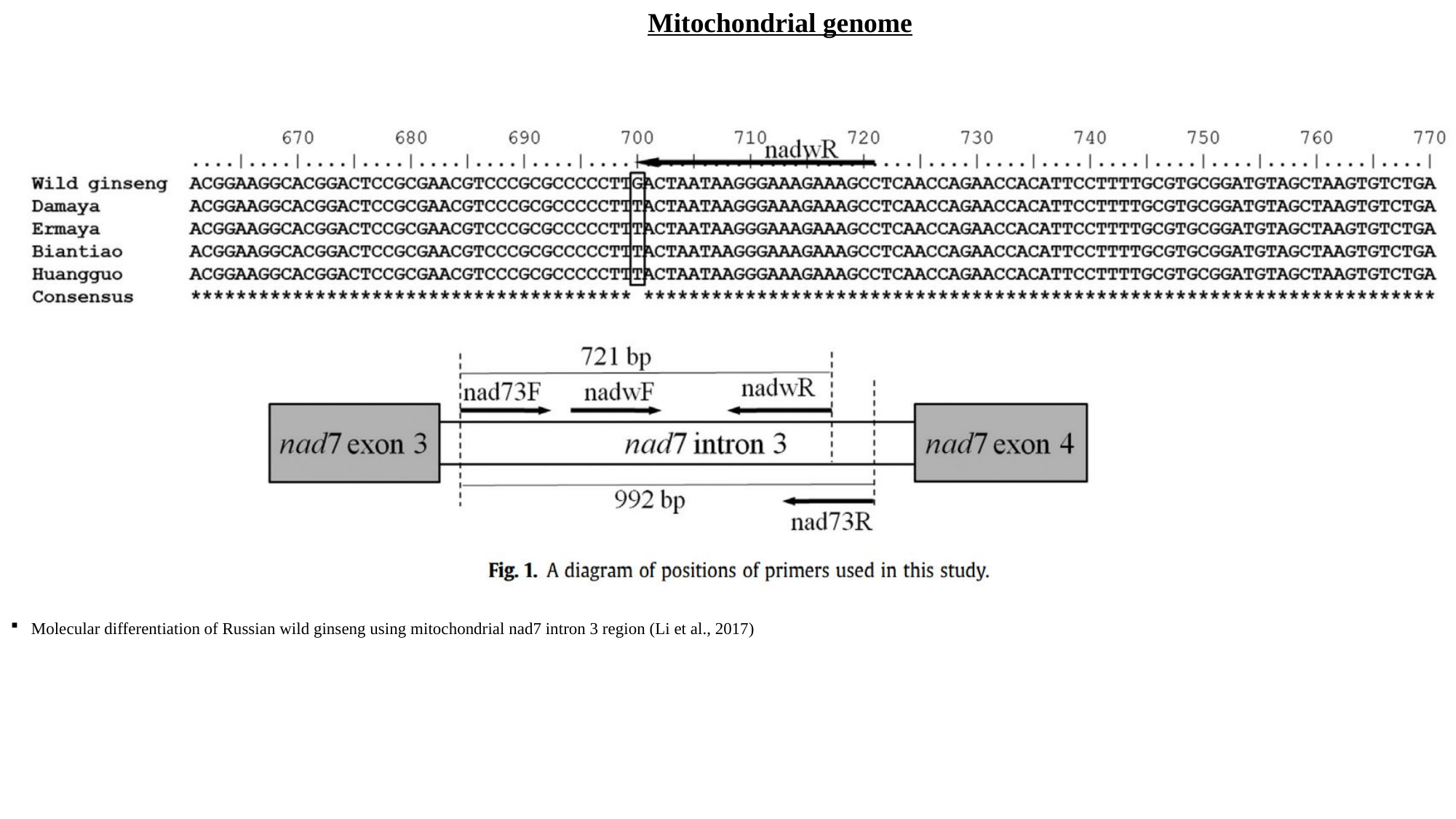

Mitochondrial genome
Molecular differentiation of Russian wild ginseng using mitochondrial nad7 intron 3 region (Li et al., 2017)

## Slide 8
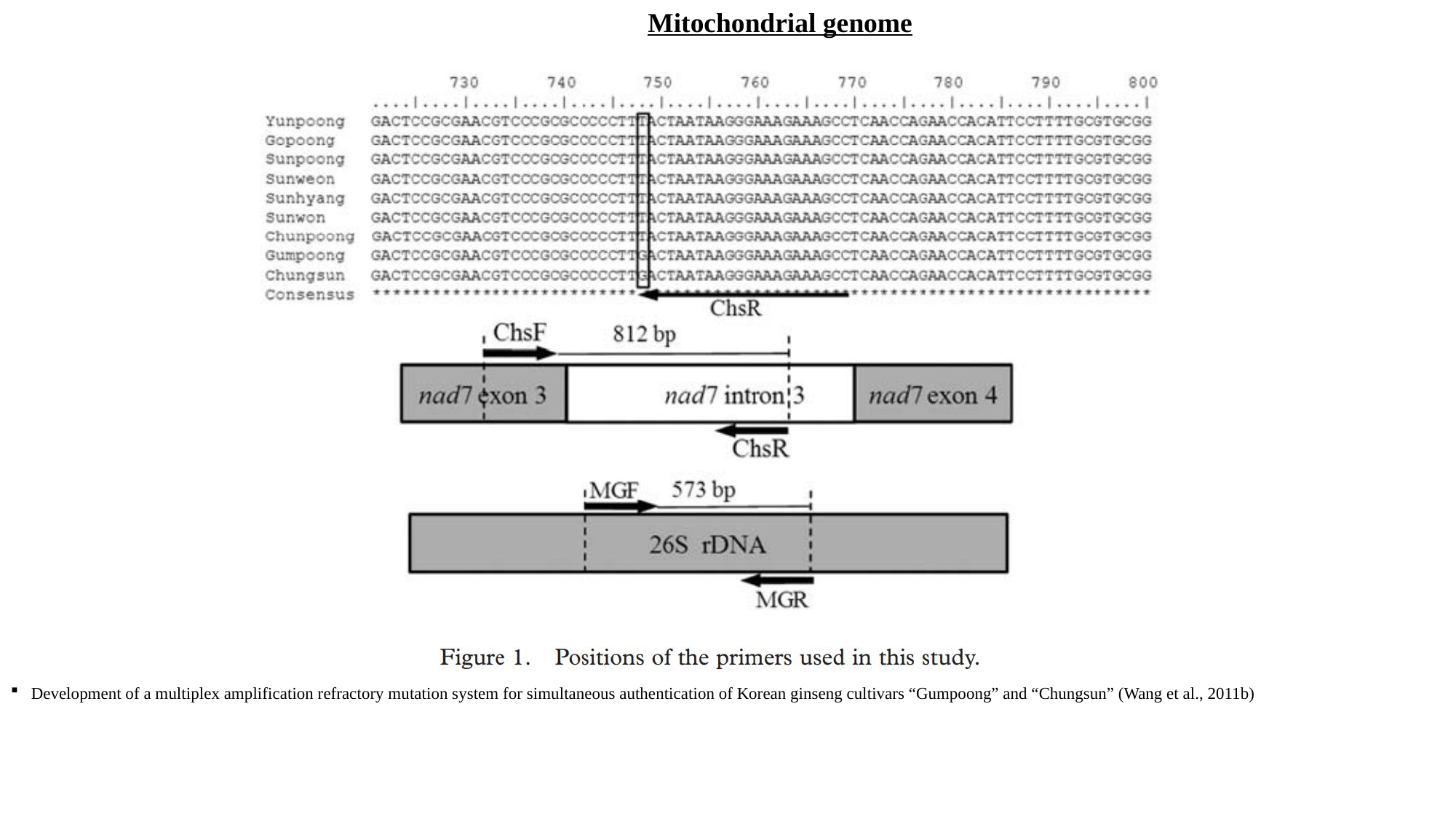

Mitochondrial genome
Development of a multiplex amplification refractory mutation system for simultaneous authentication of Korean ginseng cultivars “Gumpoong” and “Chungsun” (Wang et al., 2011b)

## Slide 9
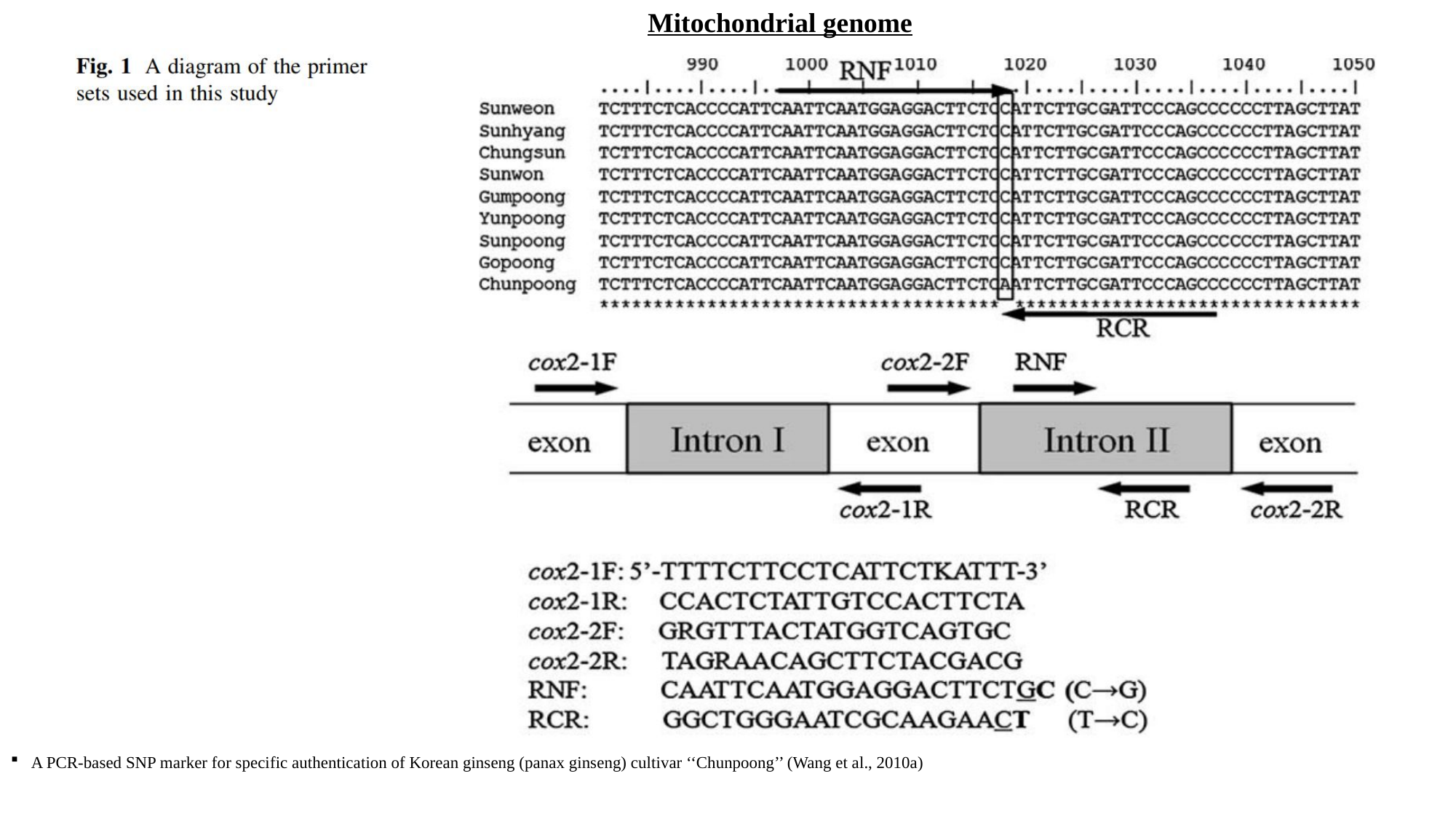

Mitochondrial genome
A PCR-based SNP marker for specific authentication of Korean ginseng (panax ginseng) cultivar ‘‘Chunpoong’’ (Wang et al., 2010a)

## Slide 10
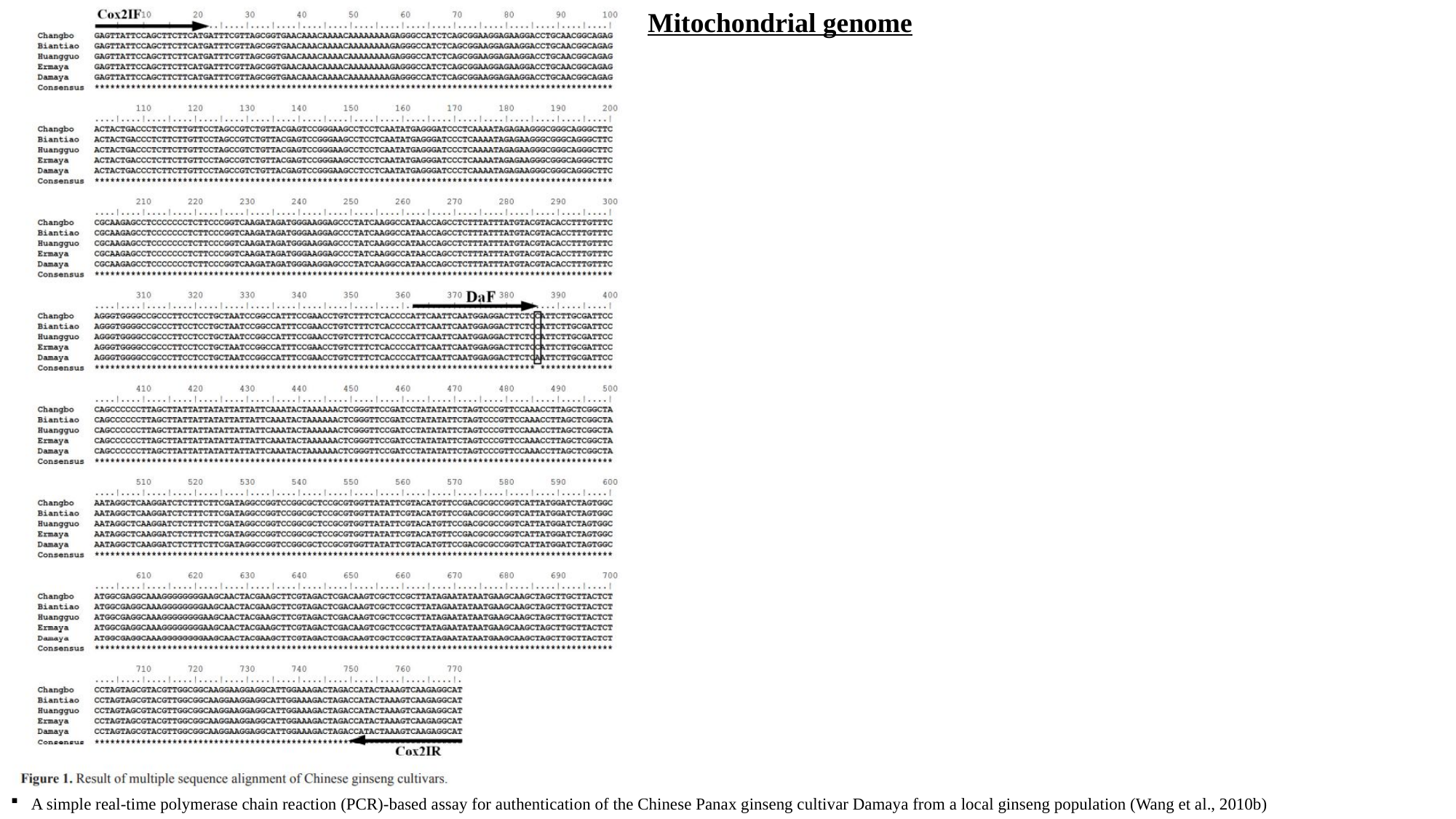

Mitochondrial genome
A simple real-time polymerase chain reaction (PCR)-based assay for authentication of the Chinese Panax ginseng cultivar Damaya from a local ginseng population (Wang et al., 2010b)

## Slide 11
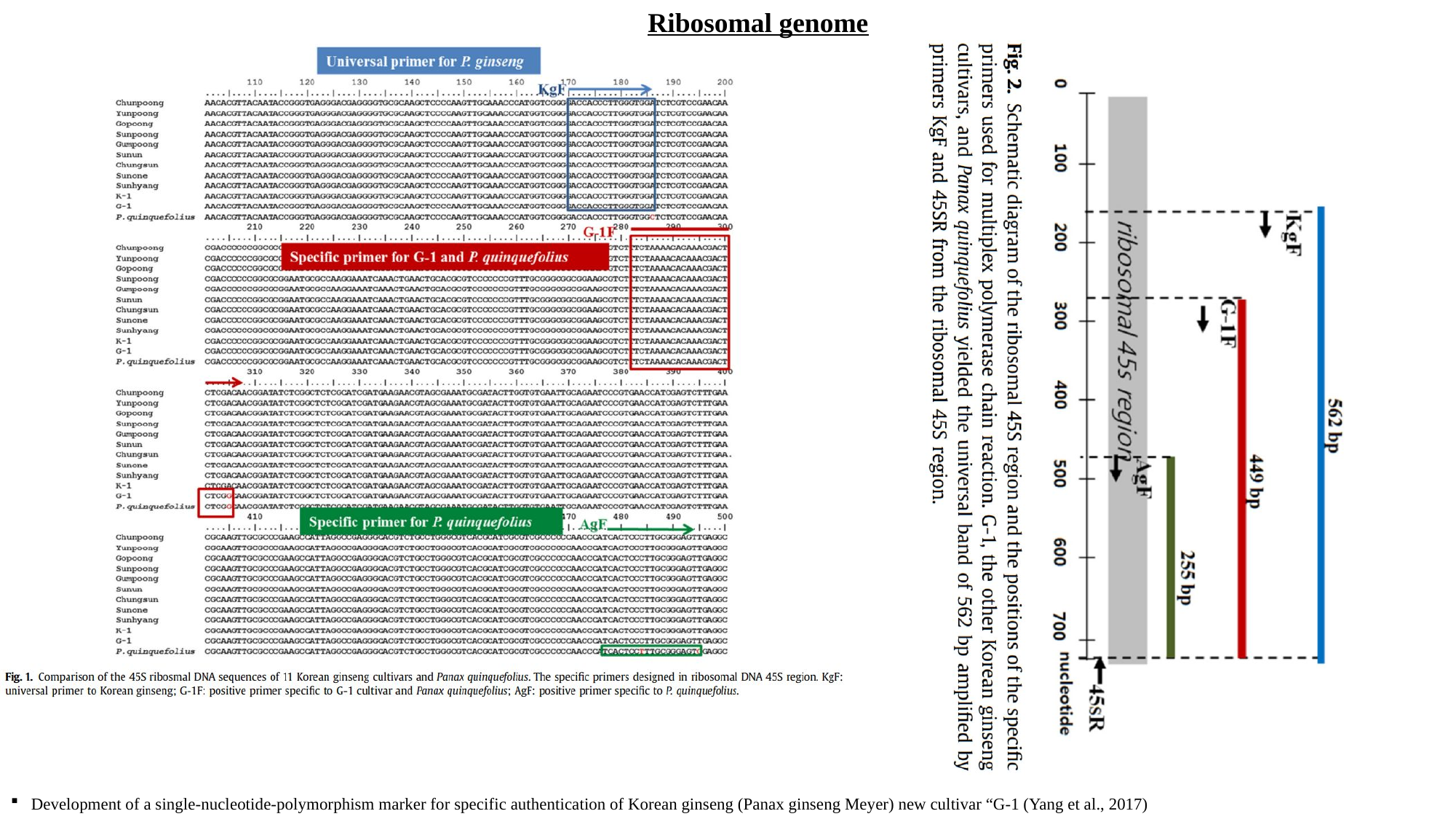

Ribosomal genome
Development of a single-nucleotide-polymorphism marker for specific authentication of Korean ginseng (Panax ginseng Meyer) new cultivar “G-1 (Yang et al., 2017)

## Slide 12
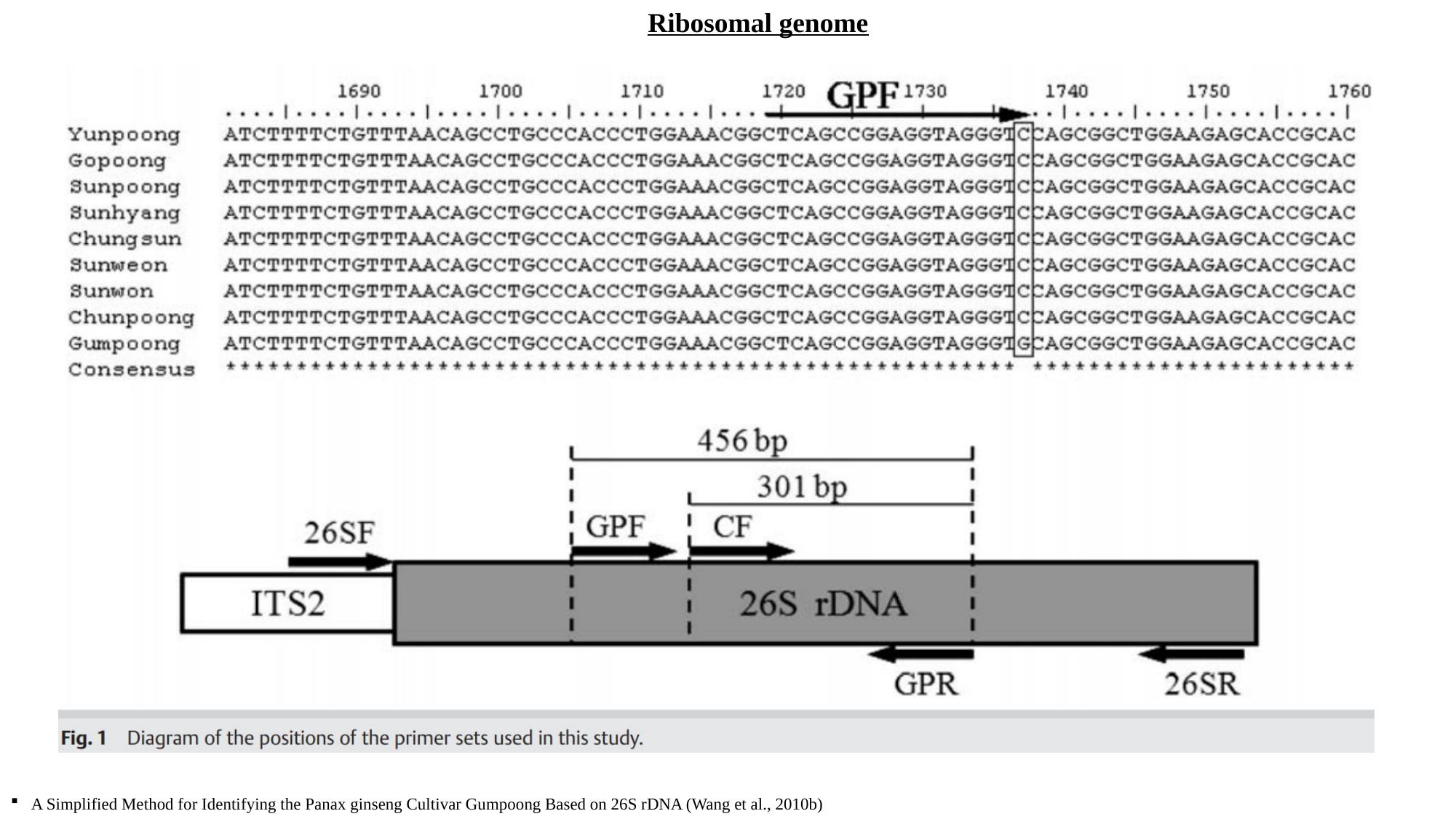

Ribosomal genome
A Simplified Method for Identifying the Panax ginseng Cultivar Gumpoong Based on 26S rDNA (Wang et al., 2010b)

## Slide 13
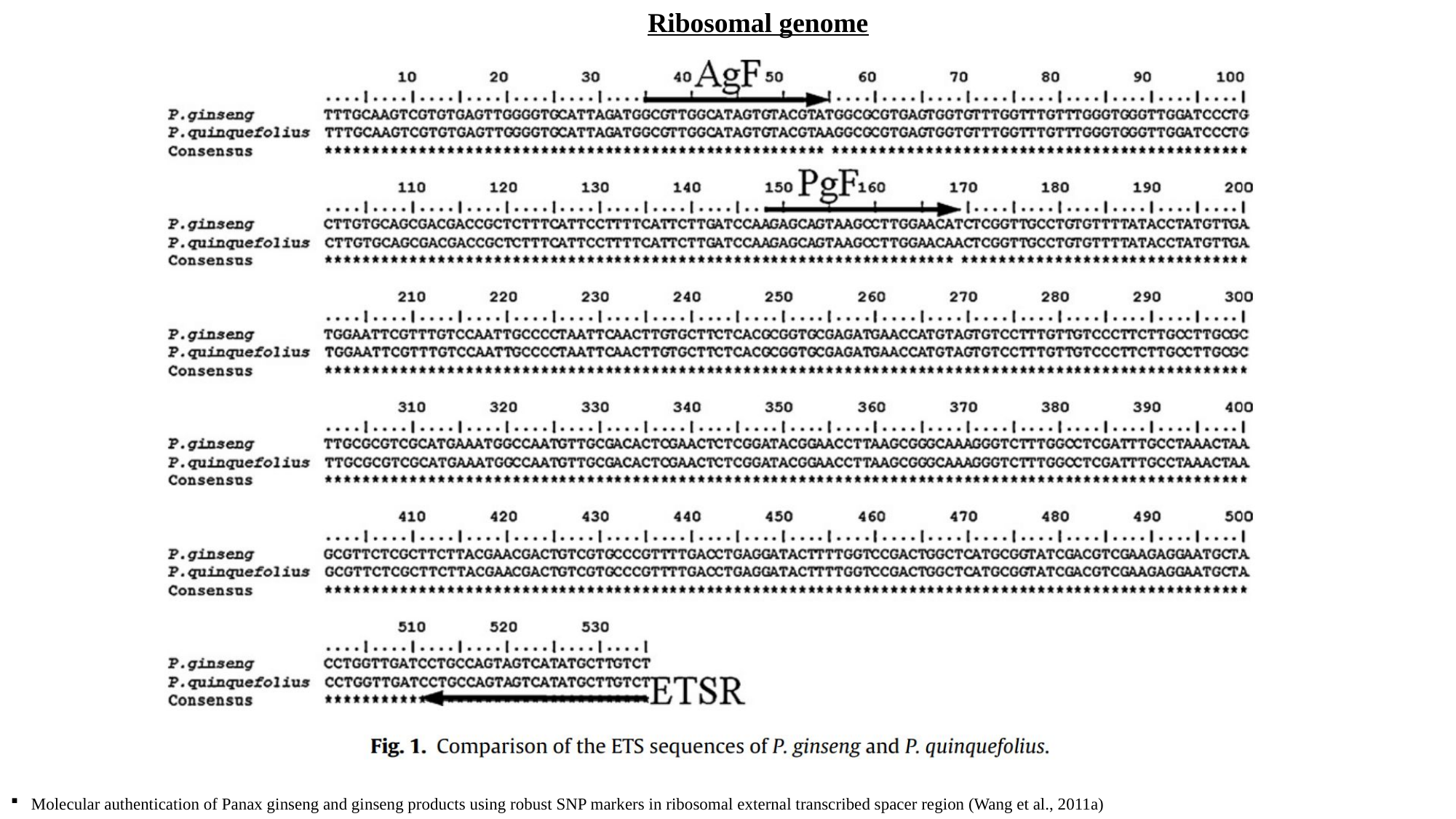

Ribosomal genome
Molecular authentication of Panax ginseng and ginseng products using robust SNP markers in ribosomal external transcribed spacer region (Wang et al., 2011a)

## Slide 14
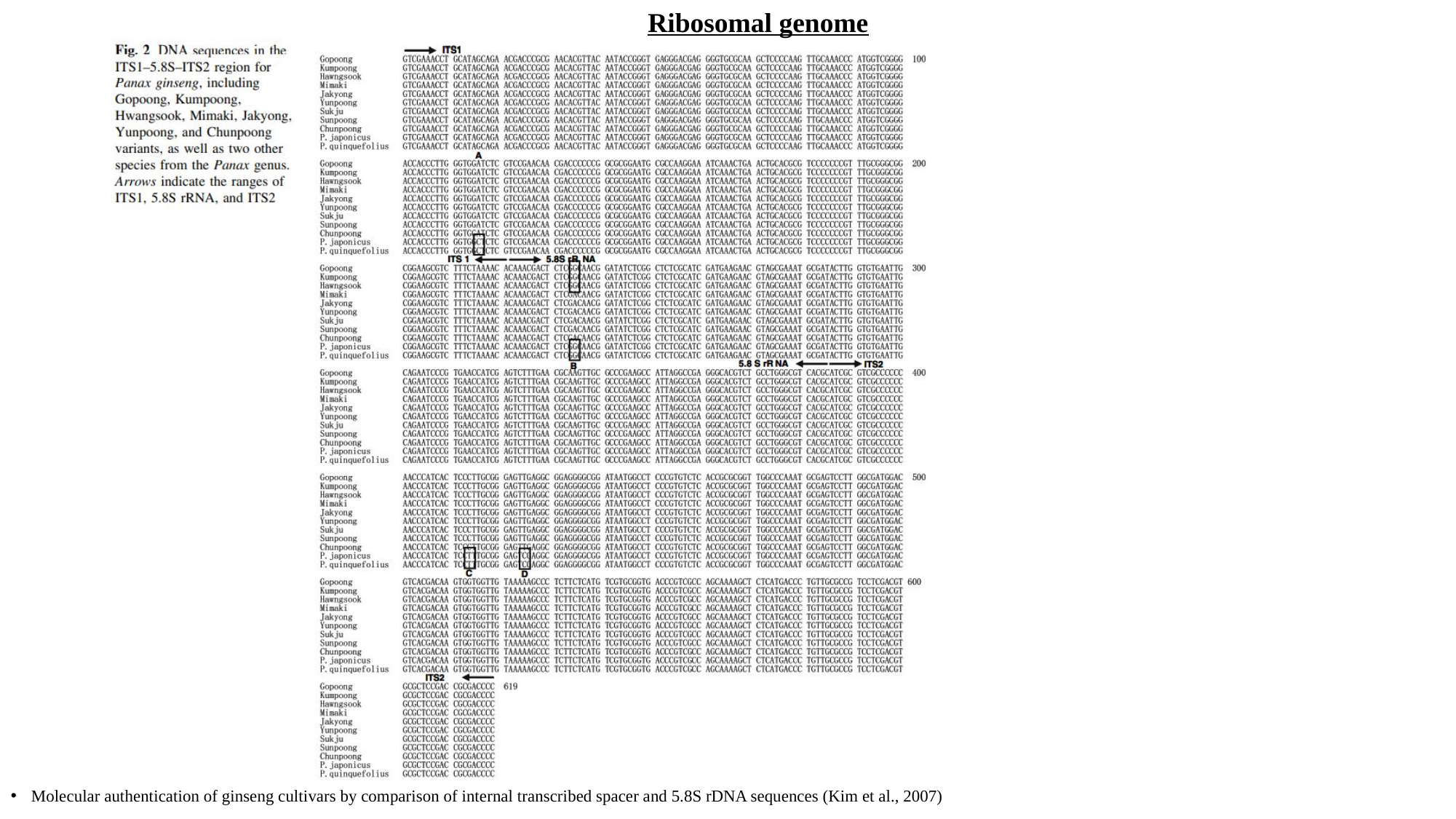

Ribosomal genome
Molecular authentication of ginseng cultivars by comparison of internal transcribed spacer and 5.8S rDNA sequences (Kim et al., 2007)

## Slide 15
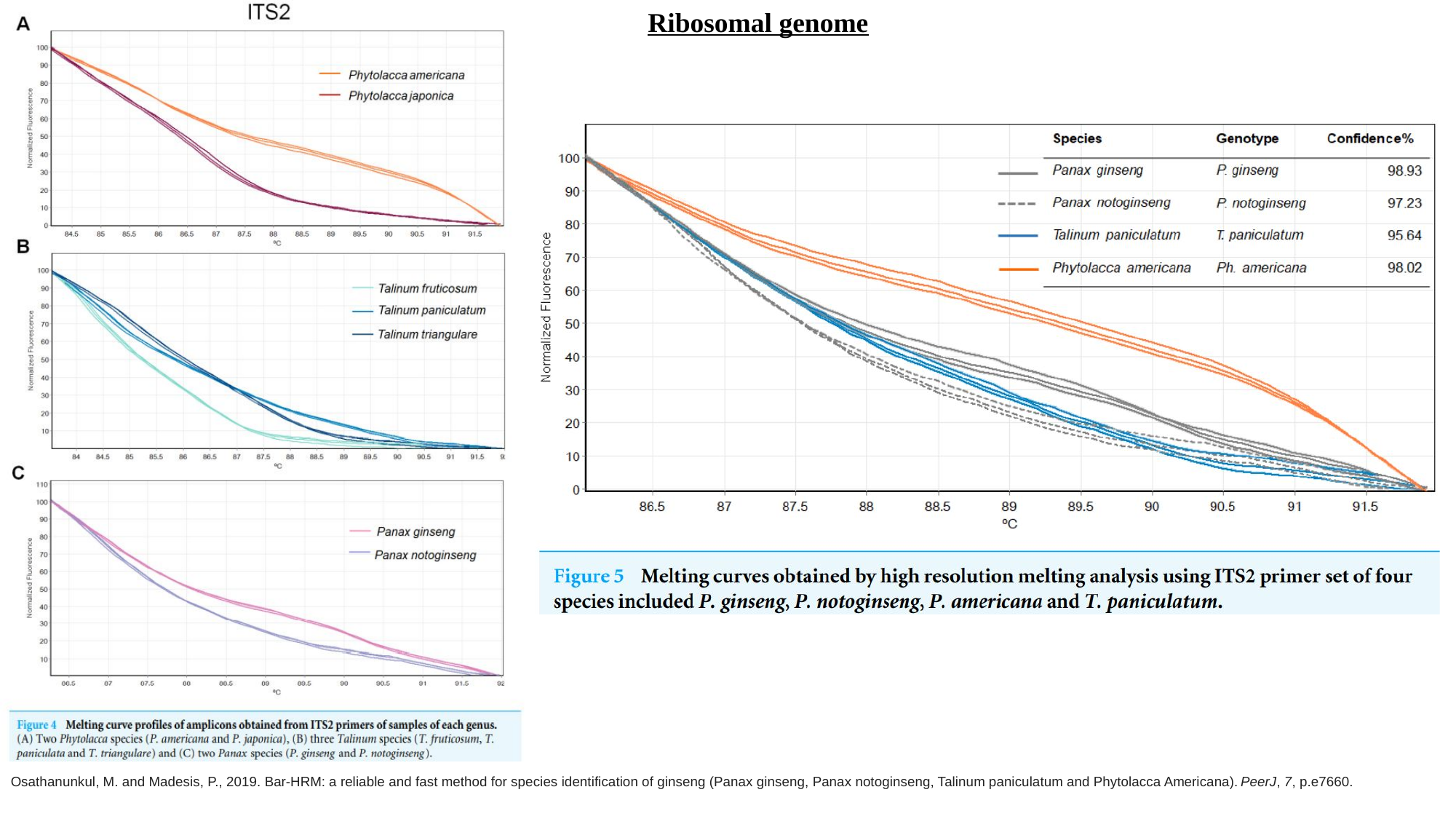

Ribosomal genome
Osathanunkul, M. and Madesis, P., 2019. Bar-HRM: a reliable and fast method for species identification of ginseng (Panax ginseng, Panax notoginseng, Talinum paniculatum and Phytolacca Americana). PeerJ, 7, p.e7660.

## Slide 16
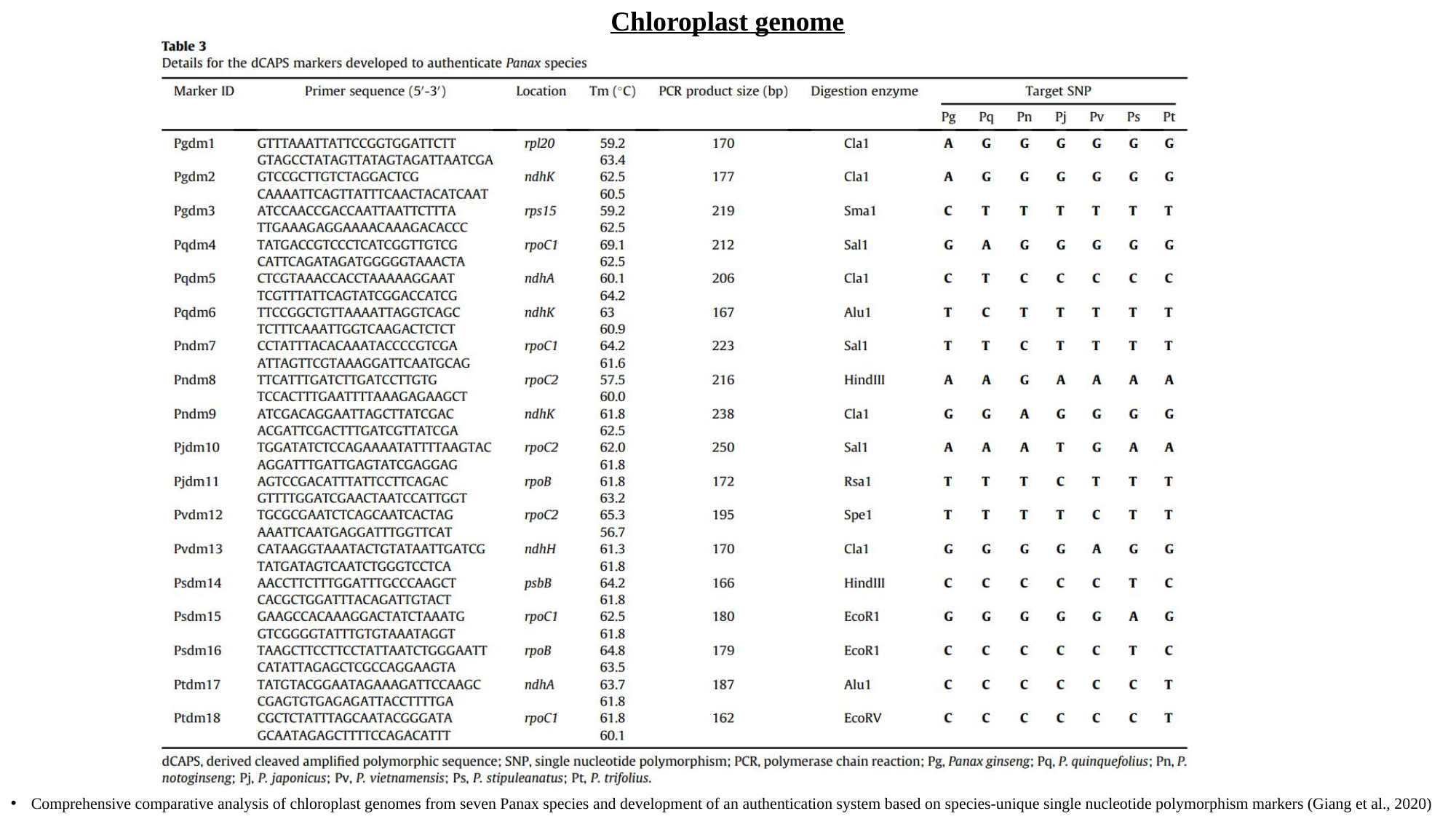

Chloroplast genome
Comprehensive comparative analysis of chloroplast genomes from seven Panax species and development of an authentication system based on species-unique single nucleotide polymorphism markers (Giang et al., 2020)

## Slide 17
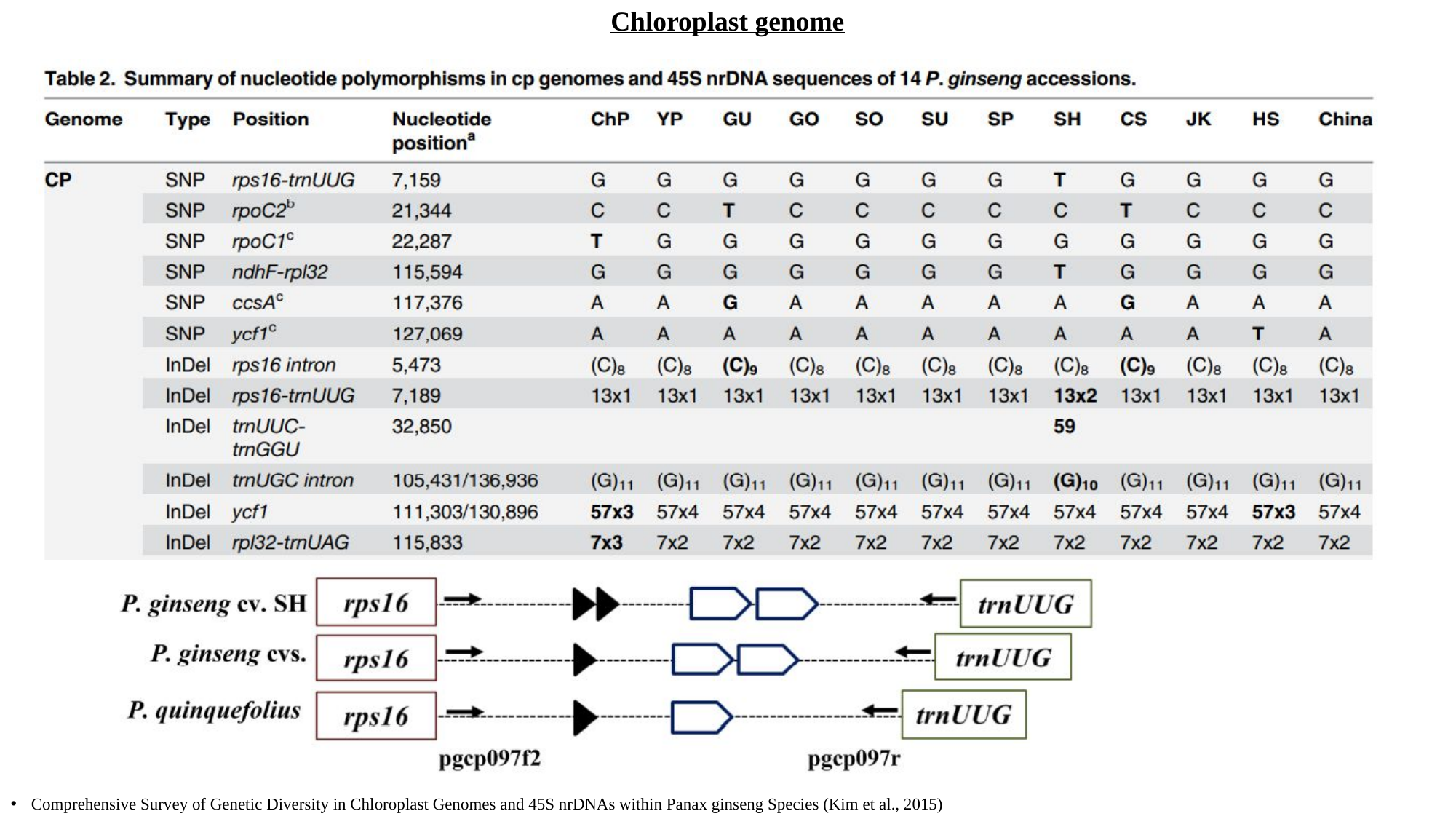

Chloroplast genome
Comprehensive Survey of Genetic Diversity in Chloroplast Genomes and 45S nrDNAs within Panax ginseng Species (Kim et al., 2015)

## Slide 18
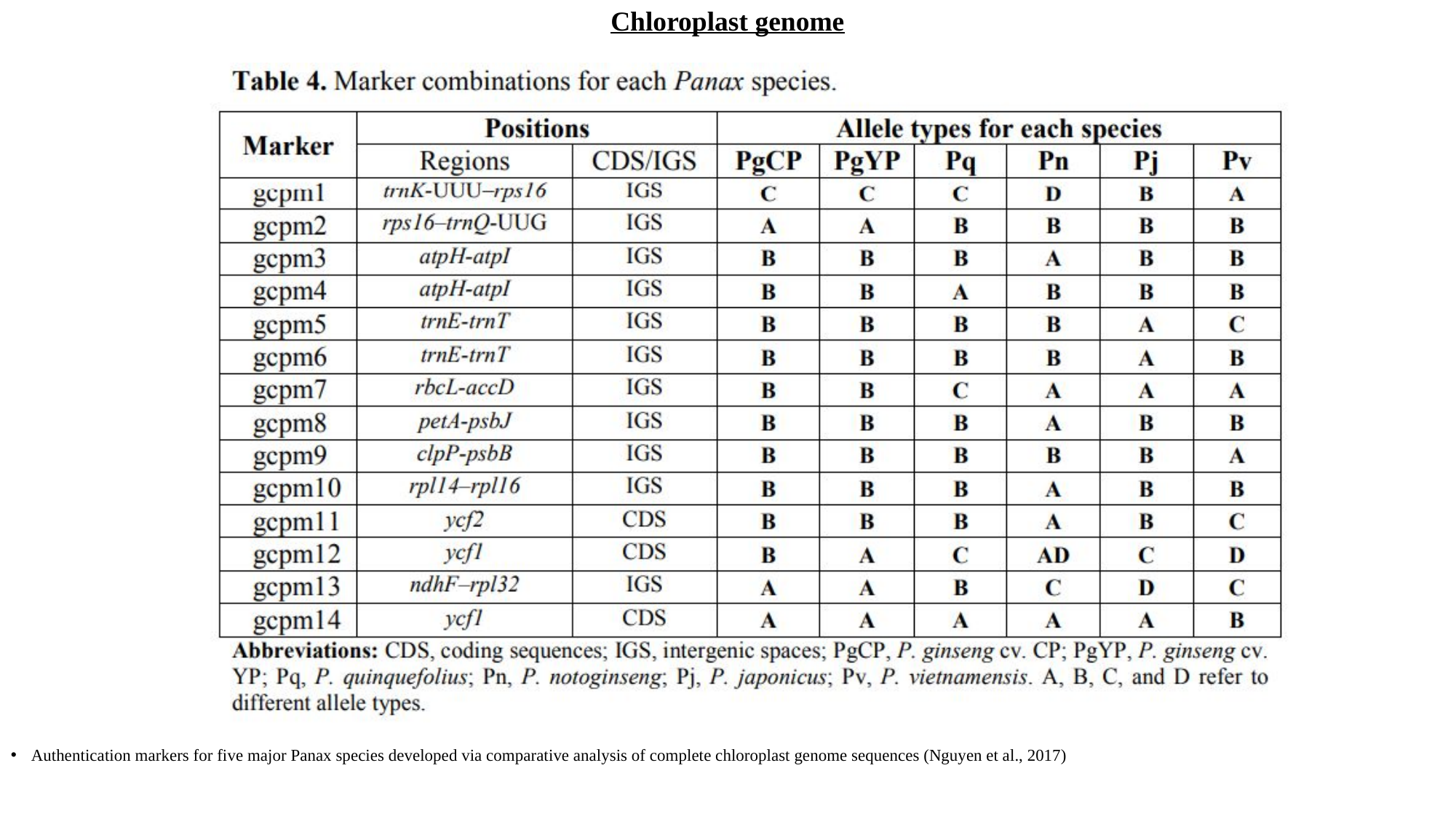

Chloroplast genome
Authentication markers for five major Panax species developed via comparative analysis of complete chloroplast genome sequences (Nguyen et al., 2017)

## Slide 19
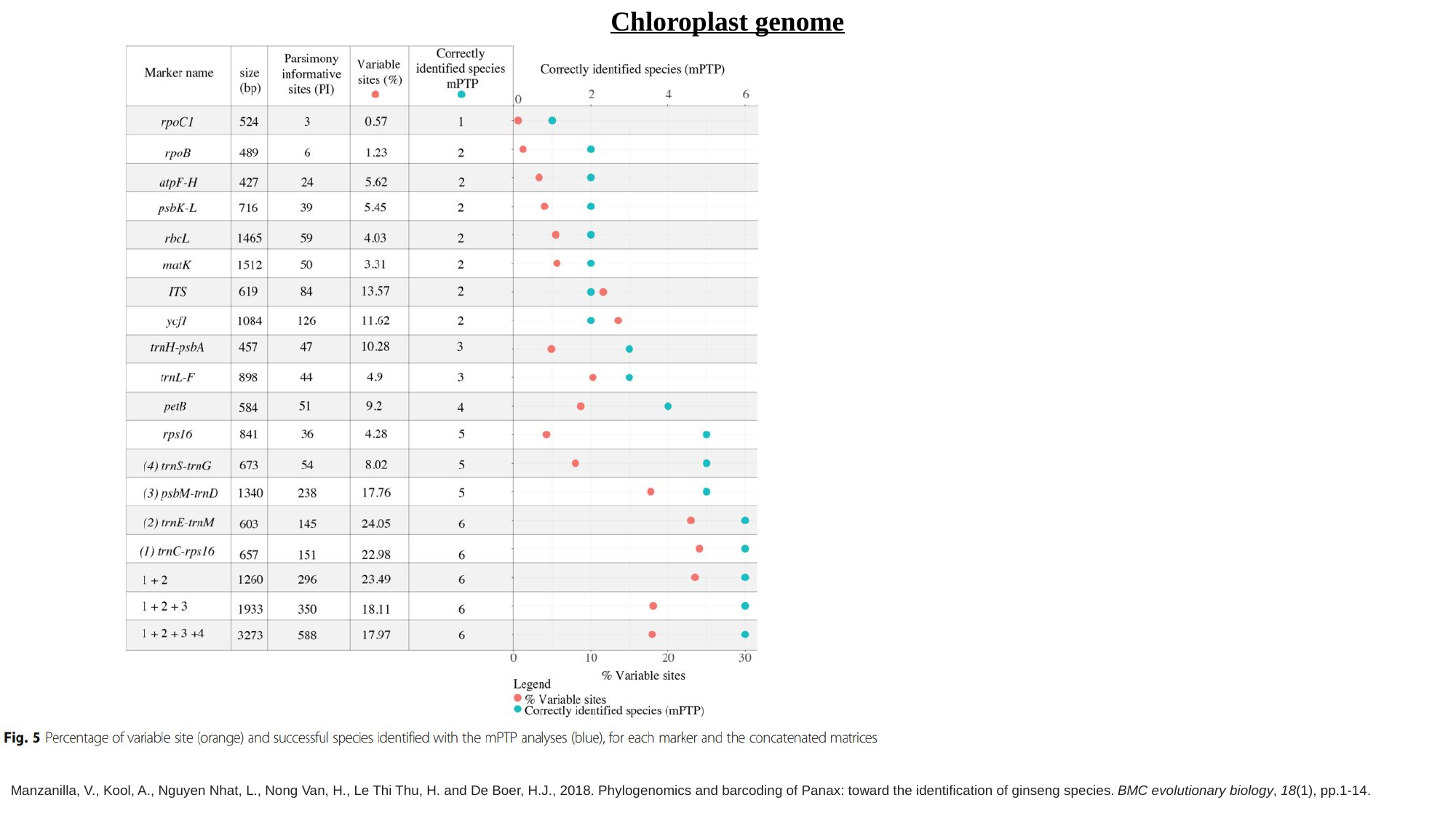

Chloroplast genome
Manzanilla, V., Kool, A., Nguyen Nhat, L., Nong Van, H., Le Thi Thu, H. and De Boer, H.J., 2018. Phylogenomics and barcoding of Panax: toward the identification of ginseng species. BMC evolutionary biology, 18(1), pp.1-14.

## Slide 20
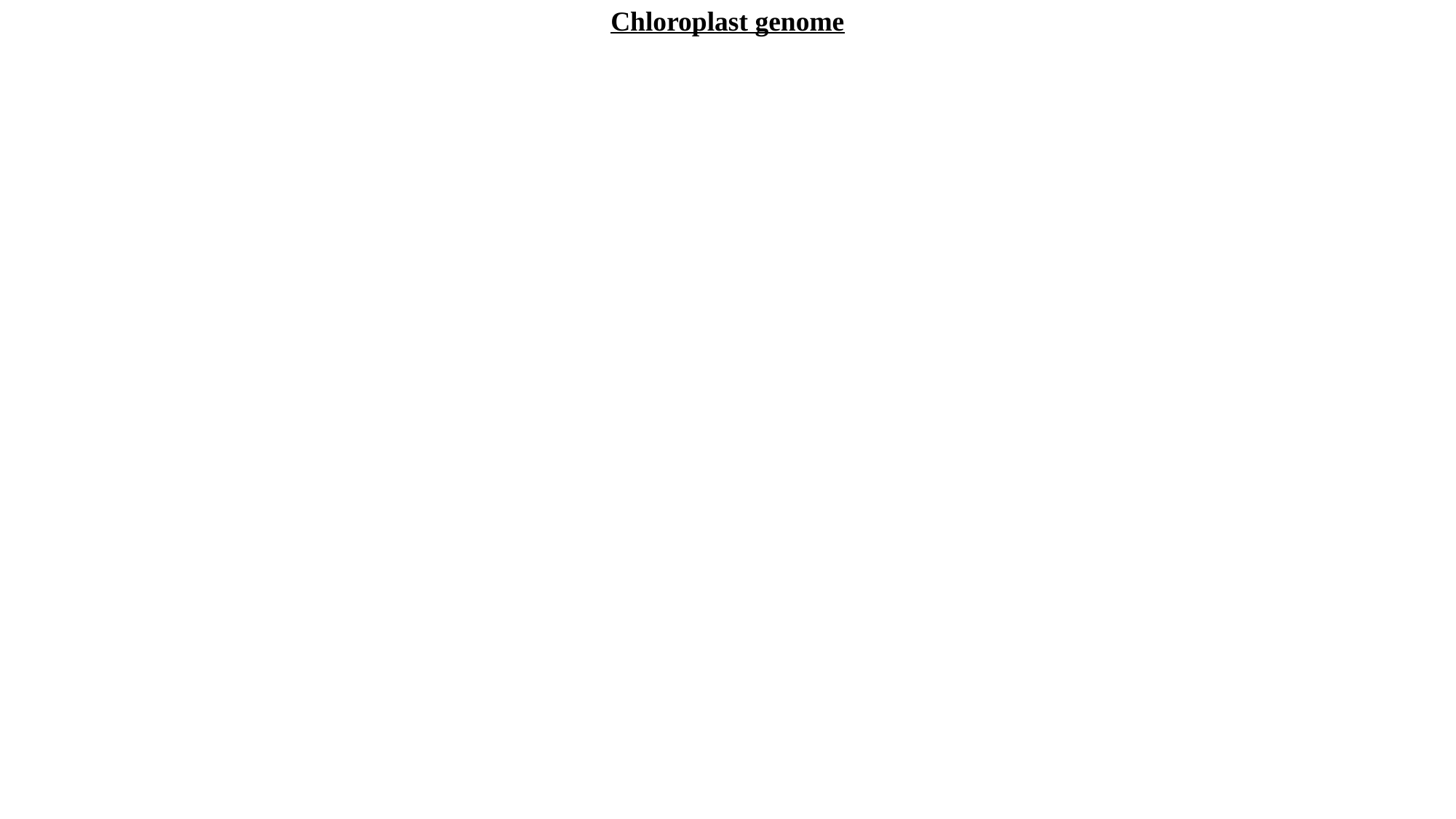

Chloroplast genome
